# Role of pyroptosis in the pathogenesis and treatment of diseases

**DOI:** 10.1002/mco2.249

**Published:** 2023-04-25

**Authors:** Xiangyu Jin, Yinchu Ma, Didi Liu, Yi Huang

**Affiliations:** ^1^ Wuxi School of Medicine Jiangnan University Jiangsu China

**Keywords:** gasdermin, inflammasome, programmed cell death, pyroptosis

## Abstract

Programmed cell death (PCD) is regarded as a pathological form of cell death with an intracellular program mediated, which plays a pivotal role in maintaining homeostasis and embryonic development. Pyroptosis is a new paradigm of PCD, which has received increasing attention due to its close association with immunity and disease. Pyroptosis is a form of inflammatory cell death mediated by gasdermin that promotes the release of proinflammatory cytokines and contents induced by inflammasome activation. Recently, increasing evidence in studies shows that pyroptosis has a crucial role in inflammatory conditions like cardiovascular diseases (CVDs), cancer, neurological diseases (NDs), and metabolic diseases (MDs), suggesting that targeting cell death is a potential intervention for the treatment of these inflammatory diseases. Based on this, the review aims to identify the molecular mechanisms and signaling pathways related to pyroptosis activation and summarizes the current insights into the complicated relationship between pyroptosis and multiple human inflammatory diseases (CVDs, cancer, NDs, and MDs). We also discuss a promising novel strategy and method for treating these inflammatory diseases by targeting pyroptosis and focus on the pyroptosis pathway application in clinics.

## Graphical abstract

This review summarizes current insights into the complicated relationship between pyroptosis and multiple inflammatory diseases, such as cardiovascular diseases (CVDs), cancer, neurological diseases (NDs), and metabolic diseases (MDs). It also discusses a promising novel strategy and method for treating these inflammatory diseases by targeting pyroptosis and focus on the pyroptosis pathway application in clinics.

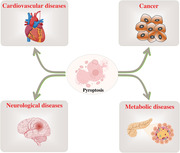

## 1 INTRODUCTION

Cell death plays an important role in maintaining homeostasis and physiological function of multicellular organism, and its abnormality is closely related to the occurrence and development of many diseases.^1^, ^2^, ^3^, ^4^, ^5^, ^6^ Based on the morphological and biochemical characteristics, cell death can be divided into two main types, accidental cell death (ACD) and programmed cell death (PCD).^7^, ^8^, ^9^, ^10^ Pyroptosis, a new type of PCD thought to be mediated by caspase‐1, was first coined by Cookson and Brennan in 2001.^11^

Morphologically, pyroptosis is mainly characterized by the formation of cell membrane pores, membrane rupture, and nuclear condensation.^3^, ^12^, ^13^, ^14^ Upon activated by inflammasome or its downstream inflammatory caspases, such as caspase‐1 in both human and mouse, caspase‐11 in mouse, and caspase‐4/5 in human, pyroptosis induces the release of intracellular content, including lactate dehydrogenase (LDH), high mobility group box 1 (HMGB1), and proinflammatory cytokines IL‐1β and IL‐18.^15^, ^16^, ^17^ As a key downstream event of inflammasome, pyroptosis plays an important role in resisting pathogen invasion.^18^, ^19^, ^20^

In recent years, emerging evidence has indicated that pyroptosis is closely related to cardiovascular diseases (CVDs),^21^, ^22^, ^23^, ^24^, ^25^ cancer,^26^, ^27^, ^28^, ^29^, ^30^, ^31^ neurological diseases (NDs),^32^, ^33^, ^34^ and metabolic diseases (MDs).^35^, ^36^, ^37^ It is also widely involved in the occurrence and progression of a variety of inflammatory diseases, especially the transformation of development of organ or tissue inflammation into cancer. In this review, we aim to summarize current insights into the molecular pathways of pyroptosis and the complicated relationship between pyroptosis and related different diseases (CVDs, cancer, NDs, and MDs). The review also provides a new idea and a promising new strategy for the prevention and treatment of diseases by targeting pyroptosis.

## 2 OVERVIEW OF PYROPTOSIS

### 2.1 The chronicle and characteristics of pyroptosis

The earliest study on pyroptosis dates back to 1986; Friedlander et al.^38^ found that treating mouse peritoneal macrophages with anthrax lethal toxin induces rapid cell death and cell contents release in an acid‐dependent manner. A subsequent study in 1992 found that the Gram‐negative bacterial pathogen *Shigella flexneri* induced the suicide of infected macrophages, which was the first time discovery of pyroptosis.^39^ However, the study did not pinpoint the type or mechanism of this cell death. In 1996, a study by Chen et al.^40^ found that the invasion plasmid antigen B (ipaB) of *S. flexneri* induces PCD of macrophages through binding directly to interleukin‐1beta converting enzyme (ICE; caspase‐1). Then, a similar study in 1998 found that the invasin SipB of *Salmonella* functions as an analog of the invasin IpaB of *Shigella* to induce infected macrophage death by binding to caspase‐1.^41^ Since some of the morphological characteristics of this form of cell death were closely similar to apoptosis, it was mistakenly considered as apoptosis at the time. However, further studies discovered that *Salmonella typhimurium* infection induced macrophage death dependent on caspase‐1 rather than classical apoptotic caspase‐3. Moreover, this form of macrophage death results in a rapid loss of membrane integrity and an unusual release of proinflammatory cytokines.^42^ These results suggest that this form of cell death is neither apoptosis nor necrosis, but a new mode of cell death. In 2001, the term of pyroptosis (from the Greek roots “pyro,” associated with fire or fever, and “ptosis” denoting a falling) was first coined by Cookson and Brennan to describe a novel form of caspase‐1‐dependent proinflammatory PCD.^11^

Pyroptosis has some morphological similarities with other types of cell death, but also has its own unique characteristics.^43^ Similar to apoptosis, pyroptosis causes DNA damage and chromatin condensation. However, pyroptosis has a unique form of DNA damage that is distinct from apoptosis. During pyroptosis, although caspase‐1 can cleave caspase‐activated DNase (CAD) in vitro,^44^ chromosomal DNA is not cleaved by CAD to produce the oligonucleosomal DNA fragments of approximately 180 bp as apoptosis, but cleaved by an unknown caspase‐1‐activated nuclease. In the meantime, their nucleuses remain intact in pyroptosis cells.^45^ In addition, membrane blebbing occurs in both apoptosis and pyroptosis.^46^ The main unique characteristics of pyroptosis is that it is mediated by members of the gasdermin protein families and caspase‐1.^47^, ^48^, ^49^ Upon infected with pathogen‐associated molecular patterns (PAMPs), such as bacterial toxins and viral nucleic acids, or stimulated by danger‐associated molecular patterns (DAMPs), such as cholesterol crystals, adenosine triphosphate (ATP), and chemotherapy drugs, caspase‐1 is activated by inflammasome, which cleaves gasdermin D (GSDMD) and induces release of its N‐terminal domain. Then, the N‐terminal domain of GSDMD transfers to the cell membrane to aggregate and form a membrane pore with an inner diameter of 10−16 nm, which enables the release of mature IL‐1β (4.5 nm) and caspase‐1 (7.5 nm).^50^ In the meantime, the extracellular water can also enter the cell through the membrane pore, causing cell swelling and eventually leading to the rupture of the cell membrane, thus releasing a large number of cell contents, including LDH and HMGB1.^51^ In addition to caspase‐1, recent studies have found that some apoptotic caspases can also trigger pyroptosis, such as caspase‐3, ‐6, and ‐8.^52^, ^53^ These studies suggested that caspase‐1 does not determine whether cells undergo pyroptosis; therefore, pyroptosis was redefined as gasdermin‐dependent PCD.

### 2.2 The executioner of pyroptosis

The name “gasdermin” comes from a mouse gene that is highly expressed in the gastrointestinal tract, especially in the esophagus and stomach.^54^ The N‐terminal sequences of the gasdermin family members are highly conservative and have the function of membrane pore formation to mediate pyroptosis.^55^ Therefore, gasdermins act as the executor of pyroptosis. Currently, there are six members of gasdermin family in human, namely GSDMA/B/C/D/E and DFNB59. Mice lacked GSMDB, but expressed three GSDMA (GSDMA1/2/3) and four GSDMC (GSDMC1/2/3/4)^56^ (Table 1).

**TABLE 1:** The types of gasdermins.

	Expression profile	Organism	Activate way	Gene function	Diseases	References
GSDMA	Upper gastrointestinal tract and skin	Human/mouse	Caspase‐3	Poorly understood	Gastric cancer, alopecia and keratosis	^54^, ^58^, ^59^, ^60^, ^61^
GSDMB	Esophageal epithelium, bladder, liver and small intestine.	Human	Caspase‐1, caspase‐3, caspase‐6, caspase‐7	Tumor‐associated gene	Skin cutaneous melanoma and bladder carcinoma	^50^, ^53^, ^66^, ^67^, ^69^
GSDMC	Spleen, skin, tonsil, small intestine and colon	Human/mouse	Caspase‐8	Melanoma metastasis‐associated gene	Melanoma, colorectal cancer	^72^, ^73^, ^76^
GSDMD	Skin, stomach, macrophage and dendritic cell	Human/mouse	Caspase‐1, caspase‐11, caspase‐4/5, caspase‐6 and caspase‐8	Pyroptosis	*Burkholderia thailandensis, Neospora caninum*	^46^, ^48^, ^49^, ^91^, ^93^
GSDME	Heart, brain, kidney, small and large intestine.	Human/mouse	Caspase‐3	Maintain hearing	Nonsyndromic hearing loss, squamous esophageal cancer, hepatocellular carcinoma, gastric cancer, colorectal cancer	^94^, ^95^, ^96^, ^97^, ^101^
DFNB59	Testis, brain, inner ear, liver and small intestine	Human/mouse	–	Maintain hearing	Nonsyndromic hearing loss	^105^, ^106^, ^107^

#### 2.2.1 GSDMA

Mouse GSDMA1 is the first member of the gasdermin family to be identified in 2000, which is specifically expressed in upper gastrointestinal tract and skin.^54^ Subsequent studies revealed that GSDMA1 had two homologous genes on chromosome 11 in mice, named GSDMA2 and GSDMA3.^57^ Similar to GSDMA1, GSDMA2 is also highly expressed in the upper region of the gastrointestinal tract in the glandular stomach, but its function is still poorly understood.^58^ GSDMA3 is specifically expressed in the hair follicle of skin, and the gain‐of‐function mutations in its C‐terminal domain have been reported to cause alopecia and keratosis.^59^, ^60^ In addition, Lei et al.^61^ found that tumor necrosis factor (TNF)‐α treatment induced significant upregulation of GSDMA3 and that it was critical for TNF‐α‐induced caspase‐3 activation and apoptosis in mouse skin keratinocytes in vivo and in vitro. Human GSDMA is relatively widely expressed, not only in the stomach and skin, but also in the pancreas esophagus and mammary gland, and it is frequently silenced in gastric cancer (GC) tissues and cells.^62^ Although GSDMA family members are highly expressed in the stomach and GSDMA3 mutations are associated with a variety of diseases, GSDMA3‐deficient mice do not have a significant phenotype under normal physiological conditions, suggesting that GSDMA may play a role in pathological conditions.^63^ Two independent research groups have recently pointed out that GSDMA, as a receptor and substrate for protease virulence factor Streptococcal pyrogenic exotoxin B (SpeB) secreted by the major human pathogen group A Streptococcus (GAS), can be cleaved by SpeB after site Gln246, thereby releasing the active amino‐terminal fragment to form lytic pores and trigger keratinocytes pyroptosis.^64^, ^65^

#### 2.2.2 GSDMB

GSDMB is the only member of the gasdermin family that is expressed only in human. Unlike GSDMA, which is restricted to the gastrointestinal tract, GSDMB is widely expressed in the esophageal epithelium, bladder, liver, and small intestine. In addition, GSDMB is expressed in a variety of tumor cells and is closely related to the occurrence and development of these tumors, skin cutaneous melanoma, and bladder carcinoma.^66^, ^67^ Consistent with GSDMA, the GSDMB can also be cleaved by caspase‐1 and its N‐terminal domain forms membrane pores.^68^ Panganiban et al.^68^ revealed that GSDMB is cleaved by caspase‐1, which releases its N‐terminal fragment and induces pyroptosis of epithelial cells, thereby promoting asthma risk. A functional splice variant rs11078928 lost 13 critical amino acids in the N‐terminal domain of GSDMB, thus inhibiting asthma by blocking pyroptosis.^68^ In addition to caspase‐1, GSDMB could be cleaved by caspase‐3, ‐6, and ‐7.^53^ However, GSDMB could not form a complete N‐terminal domain after being cleaved by caspase‐3, ‐6, and ‐7. Therefore, it remains to be further studied whether the cleaved product of GSDMB can induce pyroptosis.^50^, ^69^ Interestingly, Chen et al.^69^ found that GSDMB promotes GSDMD cleavage and noncanonical pyroptosis pathway by enhancing caspase‐4 activity, which establishes a new link between GSDMB and pyroptosis. A study by Zhou et al.^70^ found that cytotoxic T lymphocytes (CTLs) and natural killer (NK) cells‐derived granzyme A cleaves GSDMB to trigger pyroptosis in target cells. In addition to playing a crucial role in antitumor immunity, recent studies have found that the effector protein IpaH7.8 secreted by enteroinvasive *S. flexneri* targets induction of GSDMB degradation and thus inhibit NK cell bactericidal functions, suggesting that GSDMB plays an important role in defending against bacterial invasion.^71^

#### 2.2.3 GSDMC

GSDMC was first discovered as a tumor‐associated gene in 2004, which is closely associated with melanoma metastasis.^72^ In parallel, phylogenetic analysis revealed that mice contain four homologous GSDMC named GSDMC1/2/3/4, which all contain a similar N‐terminal domain.^58^ GSDMC is mainly expressed in spleen, skin, tonsil, small intestine, and colon, and studies have shown that its expression is regulated by a variety of factors.^73^ In human skin keratinocytes, ultraviolet (UV) irradiation promotes the expression of GSDMC and then increases the expression of matrix metalloproteinases‐1 (MMP‐1) through activating ERK and c‐Jun N‐terminal kinase (JNK).^74^ Further, a subsequent study by the same group found that UV light promotes GSDMC expression through the TRPV1/calcium/calcineurin/NFATc1 signaling pathway.^75^ In breast cancer, PD‐L1 acts as a transcription factor to promotes GSDMC expression by interacting with p‐STAT3 under hypoxia. Furthermore, GSDMC is specifically cleaved by caspase‐8 after treatment with TNF‐α or chemotherapy drugs, such as doxorubicin (DOX), epirubicin, and actinomycin D, thereby switching apoptosis to pyroptosis and facilitates tumor necrosis.^76^ In addition, GSDMC is also upregulated by inactivation of transforming growth factor β receptor and promoting cell proliferation in colorectal carcinogenesis.^77^ These results suggest that GSDMC is an oncogene and can be used as a therapeutic target for cancer.

#### 2.2.4 GSDMD

GSDMD is the first member of the gasdermin family to be identified as the executor of pyroptosis and the most widely studied.^48^, ^49^ GSDMD is mainly expressed in the skin, stomach, macrophage, and dendritic cell (DC).^72^ Typically, GSDMD is cleaved by inflammatory caspase‐1, a proteolytic enzyme that is activated by inflammasome in response to PAMPs and DAMPs.^78^ The crystal structure of GSDMD reveals that it is composed of two domains, among which the N‐terminal domain can form membrane pores to induce pyroptosis, and the C‐terminal domain has the self‐inhibition function to stabilize the full‐length GSDMD in the inactivated conformation.^79^, ^80^ Caspase‐1 cleaves the interdomain between the N‐terminal and C‐terminal domains of GSDMD and destroys its self‐inhibited conformation. The N‐terminal domain is released from GSDMD and subsequently transferred to the cell membrane to form pores, mediating inflammatory cytokine release, such as IL‐1β and IL‐18, and inducing pyroptosis.^79^ Interestingly, a recent study revealed that GSDMD pores‐mediated calcium influx initiates the membrane repair by recruiting the endosomal sorting complexes required for transport machinery to damaged plasma membrane areas, which enhances cell survival during pyroptosis.^81^

In addition to caspase‐1, several other caspases have also been found to cleave GSDMD and induce pyroptosis, such as caspase‐11 in mice, caspase‐4/5 in human, caspase‐6, and caspase‐8. In human monocytes, lipopolysaccharide (LPS) directly binds to the CARD domain of caspase‐4/5 and induces their activation. Subsequently, activated caspase‐4/5 cleave GSDMD and induce pyroptosis.^49^ Similarly, caspase‐11, a homologous protein of caspase‐4/5, can also be directly activated by LPS to cleave GSDMD.^48^, ^49^ It should be noted that caspase‐11 is activated not only by LPS but also by lipid A and parasite membrane glycoconjugate lipophosphoglycan.^82^, ^83^ Caspase‐8, an initiator of the extrinsic apoptosis pathway, has recently been reported by two independent groups to cleave GSDMD to induce pyroptosis during *Yersinia* infection.^84^, ^85^ Similarly, Demarco et al.^86^ also found that caspase‐8‐dependent GSDMD cleavage contributes to TNF‐induced lethality in a caspase‐1‐independent manner. Moreover, cathepsin G (CatG) and ELANE are able to proteolytically activate GSDMD.^87^, ^88^ In monocyte and neutrophil, CatG induces pyroptosis by effectively cleaving GSDMD and releasing the n‐terminal active domain, indicating that CatG is an important target for maintaining cell survival.^87^ ELANE is a neutrophil‐specific serine protease released by cytoplasmic granules in aging neutrophils, which cleaves GSDMD to induce pyroptosis.^88^

Collectively, GSDMD is not only an important executor of pyroptosis, but also mediates inflammatory cytokine release, such as IL‐1β and IL‐18, which plays a crucial role in the maintenance of homeostasis. Emerging evidence suggests that pyroptosis also plays an important role in defense against pathogen invasion and parasitic infection. Liu et al.^89^ found that the released N‐terminal domain of GSDMD can kill free bacteria in vitro and have a direct bactericidal effect in the cytoplasm of host cells. Parallel to the neutrophil extracellular trap (NET), cleaved GSDMD can also form pore‐induced intracellular trap, the remnants of a broken cell membrane that retains organelles and viable bacteria, which confines pathogen within the cellular debris of pyroptotic macrophages to defense against intracellular bacteria.^90^ Moreover, GSDMD‐mediated pyroptosis can also kill *Burkholderia thailandensis* directly in a mouse model of melioidosis.^91^ In *Brucella abortus* infection, caspase‐11 and GSDMD‐mediated pyroptosis with the help of GBP5 are critical to resist pathogen invasion.^92^ In addition, a recent study found that GSDMD protects against the intracellular parasite *Neospora caninum* infection by inducing Th1 immune response and proinflammatory cytokine secretion, including IL‐18 and IFN‐γ.^93^ Although GSDMD is involved in various pathological and physiological processes by mediating pyroptosis, whether GSDMD has an independent function of pyroptosis remains to be studied.

#### 2.2.5 GSDME

GSDME, also known as DFNA5, is highly expressed primarily in the heart, brain, kidney, and small and large intestine. GSDME was originally identified as a gene responsible for nonsyndromic hearing loss and has been implicated in a variety of tumors over the past two decades.^94^, ^95^, ^96^ DOX activates caspase‐3 and triggers GSDME‐induced pyroptosis to induce cardiac injury, GSDME plays an important role in DOX‐induced cardiac injury. Targeting Bnip3‐dependent pyroptosis pathway may be a novel therapeutic strategy to reduce DOX‐induced cardiotoxicity.^97^ In squamous esophageal cancer, patients with high GSMDE expression have a better survival rate, suggesting that GSDME can be used as a prognostic biomarker of squamous esophageal cancer.^98^ In hepatocellular carcinoma (HCC), overexpression of GSDME in HepG2 cells inhibits cell proliferation by increasing apoptosis and cell cycle arrest.^99^ In GC, transcriptional initiation region methylation of GSDME inhibits its expression in GC cell lines, and treatment of GC cells with the methylation inhibitor, 5‐aza‐2′‐deoxycytidine (5‐aza‐dC), restores the expression of GSMDE and blocks tumorigenesis.^100^ Consistent with this study, Kim et al.^101^ found that the promoter of GSDME was highly methylated (about 65%) and its expression was significantly decreased in colorectal cancer (CRC). Treatment with the methylation inhibitor 5‐aza‐dC promotes GSDME expression and inhibits tumor cell proliferation and tumorigenesis, suggesting GSDME as a novel tumor suppressor gene in CRC.^101^ Moreover, recent studies have found that GSDME plays an important role in pyroptosis. During chemotherapy, caspase‐3 cleaves GSDME after Asp270 to release its N‐terminal domain, which has the ability to form membrane pores and eventually leads to cell pyroptosis.^52^ In addition to chemotherapy, some other apoptotic triggers, such as etoposide or vesicular stomatitis virus infection, can also induce caspase‐3 activation and GSMDE cleaving, causing secondary necrosis/pyroptosis.^102^ Interestingly, GSDME not only form pores in the plasma membrane, but also mediates mitochondrial pore formation, which induces cytochrome *C* release and caspase‐3 activation in response to apoptotic stimulis.^103^ In addition to caspase‐3, a recent study by Zhang et al.^104^ found that GSDME can also be cleaved and activated by granzyme B in a caspase‐independent manner, revealing that tumor‐infiltrating NK and CD8^+^ T lymphocytes inhibit carcinogenesis by inducing tumor cells pyroptosis.

#### 2.2.6 DFNB59

DFNB59, a long‐neglected member of the gasdermin family, has received little coverage so far. Like GSDME, DFNB59 truncation mutations also cause cochlear hearing impairment and central vestibular dysfunction.^105^ DFNB59 is mainly expressed in testis, brain, inner ear, liver, and small intestine, and its primary function is to maintain hearing.^106^ Previous clinical studies have identified that a c.406C>T (p.R136X) nonsense mutation in the DFNB59 gene is associated with autosomal recessive nonsyndromic hearing loss.^107^ Schwander et al.^108^ shown through a forward genetics screen that a 122delA mutation in the DFNB59 gene causes outer hair cell (OHC) defects and hearing loss by introducing a premature stop codon in human. Moreover, another study by Delmaghani et al.^106^ found that the R183W variant of DFNB59 causes nonsyndromic deafness was associated with neuronal defect. In addition, DFNB59 was found to interact with the coiled‐coil domains of ROCK2, an effector of the small GTPase Rho, and the scaffold protein IQGAP1 through its C‐terminal domain, both are well‐known actin/microtubule dynamics regulators that alter cell shape and contribute to DFNB59 to maintain the function of OHCs in a cellular autonomous manner.^109^ However, the C‐terminal domain of DFNB59 is extremely short compared with other members of the gasdermin protein family, it is unclear whether the extremely short C‐terminal domain of DFNB59 has the same self‐inhibitory function as other gasdermin proteins, and whether the N‐terminal domain of DFNB59 has the ability of membrane pore formation is also unknown, which requires further investigate.

### 2.3 The signaling pathways of pyroptosis

**FIGURE 1:**
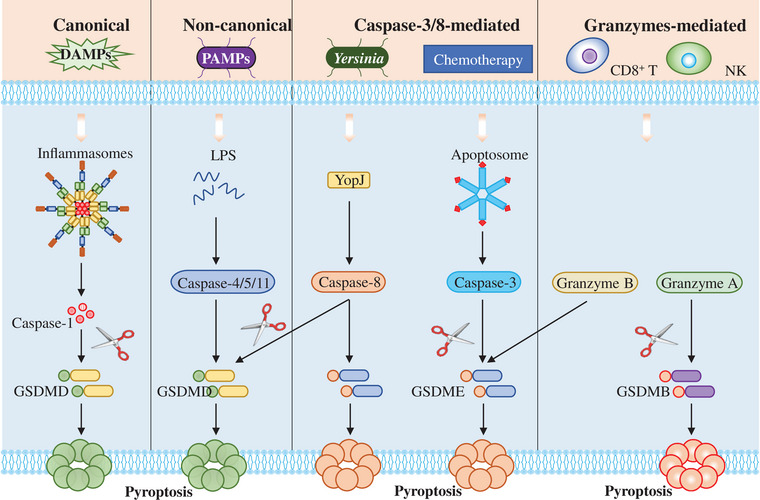
The activation of pyroptosis pathways. In canonical pyroptosis pathway, NLRP3 recruits the adaptor protein ASC and procaspase‐1 to assemble into inflammasome in response to large amounts of PAMPs and DAMPs, which induces procaspase‐1 self‐cleavage into mature caspase‐1. Then, the activated caspase‐1 cleaves gasdermin D (GSDMD) and releases its N‐terminal domain to form membrane pores and inducing pyroptosis. In noncanonical pyroptosis pathway, pyroptosis is induced by murine caspase‐11 or the human homologue caspase‐4/‐5, which can be activated by LPS from Gram‐negative bacteria. In caspase‐3/8‐mediated pyroptosis pathway, the effector protein YopJ from *Yersinia* promotes the activation of caspase‐8 by inhibiting TAK1 kinase. Then, the activated caspase‐8 cleaves GSDMD as well as GSDME to induce pyroptosis. In addition, caspase‐3 activated by chemotherapy drugs can also cleave GSDME to induce pyroptosis. In granzymes‐mediated pyroptosis pathway, granzyme A and granzyme B from tumor‐infiltrating CD8^+^ T lymphocytes and natural killer (NK) can directly cleave GSDME and GSDMB respectively to induce pyroptosis.

Gasdermins are the executor of pyroptosis, and many proteolytic enzymes have been reported to be able to cleave gasdermins to induce pyroptosis. To date, pyroptosis is mainly mediated by four signaling pathways, namely canonical pyroptosis pathway, noncanonical pyroptosis pathway, other caspases‐mediated pyroptosis pathway and granzymes, and other proteases‐mediated pyroptosis pathway (Figure 1).

#### 2.3.1 Canonical pyroptosis pathway

Canonical pyroptosis is induced by caspase‐1, which is activated by inflammasome in response to PAMPs and DAMPs. So far, five main types of pattern recognition receptors (PRRs) have been identified, including Toll‐like receptors (TLRs), C‐type lectin receptors (CLRs), retinoic acid‐inducible gene‐I‐like receptors (RLRs), cytoplasmic DNA sensors and nucleotide‐binding, and oligomerization domain‐like receptors (NLRs).^110^, ^111^ These PRRs are expressed on both immune cells and nonimmune cells. After recognizing their corresponding ligands, PRRs initiate and activate a variety of innate immune signaling pathways, produce a series of cytokines that promote inflammation and mediate immune responses. TLRs are the most deeply studied class of PRRs. TLRs can recognize a variety of pathogen‐related molecular patterns, including LPS and flagellin on the surface of bacteria, viral single‐stranded RNA (ssRNA), double‐stranded RNA (dsRNA), and fungi.^112^ CLRs family contains 17 subtypes. CLRs can recognize a series of ligands, including carbohydrates, and participate in a variety of physiological processes of the body.^113^ RLRs include retinoic‐acid inducible gene I (RIG‐I), melanoma differentiation‐associated 5 (MDA5), and laboratory of genetics and physiology 2 (LGP2). RIG‐I and MDA5 recognize viral RNA in the cytoplasm and then produce type I interferons through downstream signals to initiate the host antiviral response.^114^ The two most important DNA receptors in the cytoplasm are AIM2 and cGAS. AIM2 inflammasome is essential for cells to resist the invasion of pathogens such as DNA viruses and bacteria,^115^ and cGAS–STING signaling pathway plays an important role in autoimmune diseases, tumors, and host defense.^116^ NLRs are a class of intracellular PRRs. NLRs are widely expressed in various immune cells and epithelial cells, and can initiate innate immune response by recognizing intracellular PAMPs and DAMPs.^117^ Inflammasome is a polymeric protein complex composed of PRRs, adaptor proteins ASC, and effector protein procaspase‐1.^118^ To date, five PRRs are widely accepted to form inflammasome, including NLRP1, NLRP3, NLRC4, AIM2, and pyrin.^119^ In general, inflammasome activation requires two steps. During the prestimulation step, some bacterial components, such as LPS, induce the expression of PRRs and pro‐IL‐1β through TLR4–NF‐κB pathway. In the subsequent assembly and activation step, the PRRs sensor molecules recruit ASC and procaspase‐1 to form inflammasome through interaction upon stimulated by PAMPs and DAMPs^120^ (Table 2). The assembled inflammasome induced procaspase‐1 self‐cleavage and activation. Then, the activated caspase‐1 cleaves gasdermin D (GSDMD) and releases its N‐terminal domain, allowing its oligomerization to form membrane pores and inducing pyroptosis, which not only contribute to proinflammatory cytokine secretion and cell contents release, but also crucial for host defense against pathogens and maintain homeostasis.^121^

**TABLE 2:** DAMPs, PAMPs, and their corresponding PRRs.

PRR	PAMPs	DAMPs	References
TLRs	Viruses, bacteria, fungi	HMGB1, mRNA, microRNAs	^112^
CLRs	Fungi	F‐actin, β‐glucosylceramide	^113^
RLRs	Viruses	Endogenous 5′ppp RNA, endogenous retroviral RNA	^114^
CDSs	Microbial DNA	Cytoplasmic DNA, damaged DNA	^115^, ^116^
NLRs	Viruses, bacteria, fungi	MSU, glucose, cholesterol crystals, Aβ, ATP	^117^

#### 2.3.2 Noncanonical pyroptosis pathway

In the noncanonical pyroptosis pathway, pyroptosis is induced by murine caspase‐11 or the human homologue caspase‐4/‐5, which is activated by LPS from Gram‐negative bacteria.^82^ Mechanically, LPS or lipid A directly bind to the N‐terminal CARD domain of caspase‐11 or caspase‐4/‐5 with high specificity and affinity, which leads to their oligomerization and activation. resulting in GSDMD cleavage, inducing cell membrane pore formation and pyroptosis.^82^, ^122^ Similar to caspase‐1, although caspase‐11 or caspase‐4/‐5 can cleave GSDMD and cause cell membrane pore formation and pyroptosis, it cannot directly cleave pro‐IL‐1 and pro‐IL‐18.^122^ Interestingly, caspase‐11‐mediated noncanonical pyroptosis induces potassium outflow, which leads to inflammasome activation and proinflammatory cytokines maturation and secretion.^123^ In addition to reducing the intracellular potassium level, Yang et al.^124^ recently found that caspase‐11 can also cleave pannexin‐1 to promote ATP release, which in turn facilitates inflammasome activation and proinflammatory cytokine secretion.

#### 2.3.3 Other caspases‐mediated pyroptosis pathway

Similar to caspase‐1, caspase‐11, and caspase‐4/‐5, several other caspases have been reported to cleave gasdermin to induce pyroptosis. Caspase‐8, an initiator of the extrinsic apoptosis pathway, has recently been reported by two independent groups to cleave gasdermin to induce pyroptosis. In response to *Yersinia* infection, its effector protein YopJ promotes caspase‐8 to cleave GSDMD by inhibiting TAK1 or IκB kinase (IKK).^84^ Similarly, Sarhan et al.^85^ found that caspase‐8 was activated by costimulation of LPS and (5Z)‐7‐Oxozeaenol, a small‐molecule inhibitor of TAK1, and subsequently the activated caspase‐8 cleaves GSDMD to induce pyroptosis in murine macrophages. Consistent with this finding, a recent study by Demarco et al.^86^ found that caspase‐8‐dependent GSDMD cleavage also contribute to TNF‐induced lethality in a caspase‐1‐independent manner. Caspase‐3, an apoptotic caspase that activated by TNF‐α or chemotherapy drugs, specifically cleaves GSDME in its linker, releasing its N‐terminal domain to form membrane pores to induce pyroptosis.^52^, ^125^ Moreover, Zheng et al.^126^ found that caspase‐6 facilitates ZBP1‐mediated inflammasome activation and pyroptosis in response to influenza A virus infection. Notably, a recent study found that caspase‐6 directly cleaves caspase‐11 at Asp59 and Asp285 to induce pyroptosis during Gram‐negative bacteria infection.^127^

#### 2.3.4 Granzymes and other proteases‐mediated pyroptosis pathway

Granzymes are specific cytotoxic lymphocyte granulation associated with serine proteases that have been implicated in lymphocyte functions to protects organisms against viral infection and cellular transformation.^128^ Recent studies have found that granzymes are also closely related to pyroptosis.^70^, ^104^ Granzyme B, derived from tumor‐infiltrating CD8^+^ T lymphocytes and NK, directly cleaves GSDME at the same site as caspase 3 to induce pyroptosis of target cells, thereby enhancing antitumor immunity and act as a tumor suppressor.^104^ Moreover, Zhou et al.^70^ found that another lymphocyte‐derived protease granzyme A can also induce pyroptosis by cleaving GSDMB at the site Lys229/Lys244. In addition to granzymes, several other proteases have been found to mediate pyroptosis, such as CatG and elastase (ELANE).^87^, ^88^ In monocyte and neutrophil, CatG induces pyroptosis by effectively cleaving GSDMD and releasing the n‐terminal active domain, indicating that CatG is an important target for maintaining cell survival.^87^ ELANE is a neutrophil‐specific serine protease released by cytoplasmic granules in aging neutrophils, which cleaves GSDMD to induce pyroptosis.^88^

## 3 PYROPTOSIS AND CVDs

**FIGURE 2:**
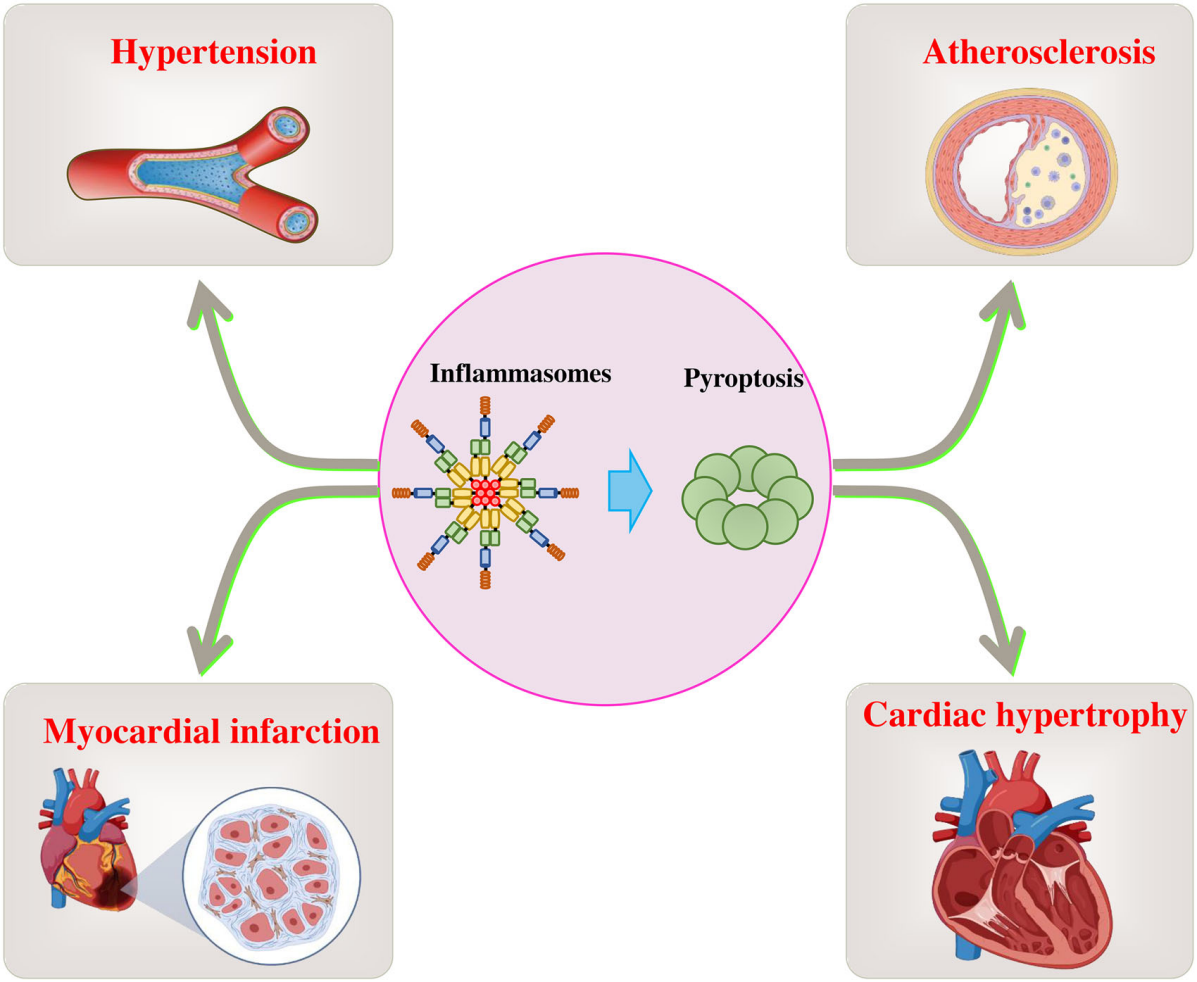
Pyroptosis promotes the occurrence of multiple cardiovascular diseases. Inflammasome‐mediated pyroptosis is involved in the pathogenesis processes of multiple cardiovascular diseases, including atherosclerosis, myocardial infarction (MI), hypertension, and cardiac hypertrophy.

CVDs are an umbrella term for disorders of the heart and blood vessels, including atherosclerosis, myocardial infarction, hypertension, diabetic cardiomyopathy (DCM), myocarditis, and cardiac hypertrophy, which are the leading cause of death globally, killing more than 17 million people each year.^129^ Although there have been significant improvements in the treatment of CVDs, the development of novel and effective therapies remains a major research goal in this field. In the past decades, studies have shown that the occurrence of CVDs is closely related to cell death, suggesting that targeting cell death is an effective intervention for the treatment of CVDs^130^, ^131^, ^132^, ^133^ (Figure 2).

### 3.1 Pyroptosis and atherosclerosis

**FIGURE 3:**
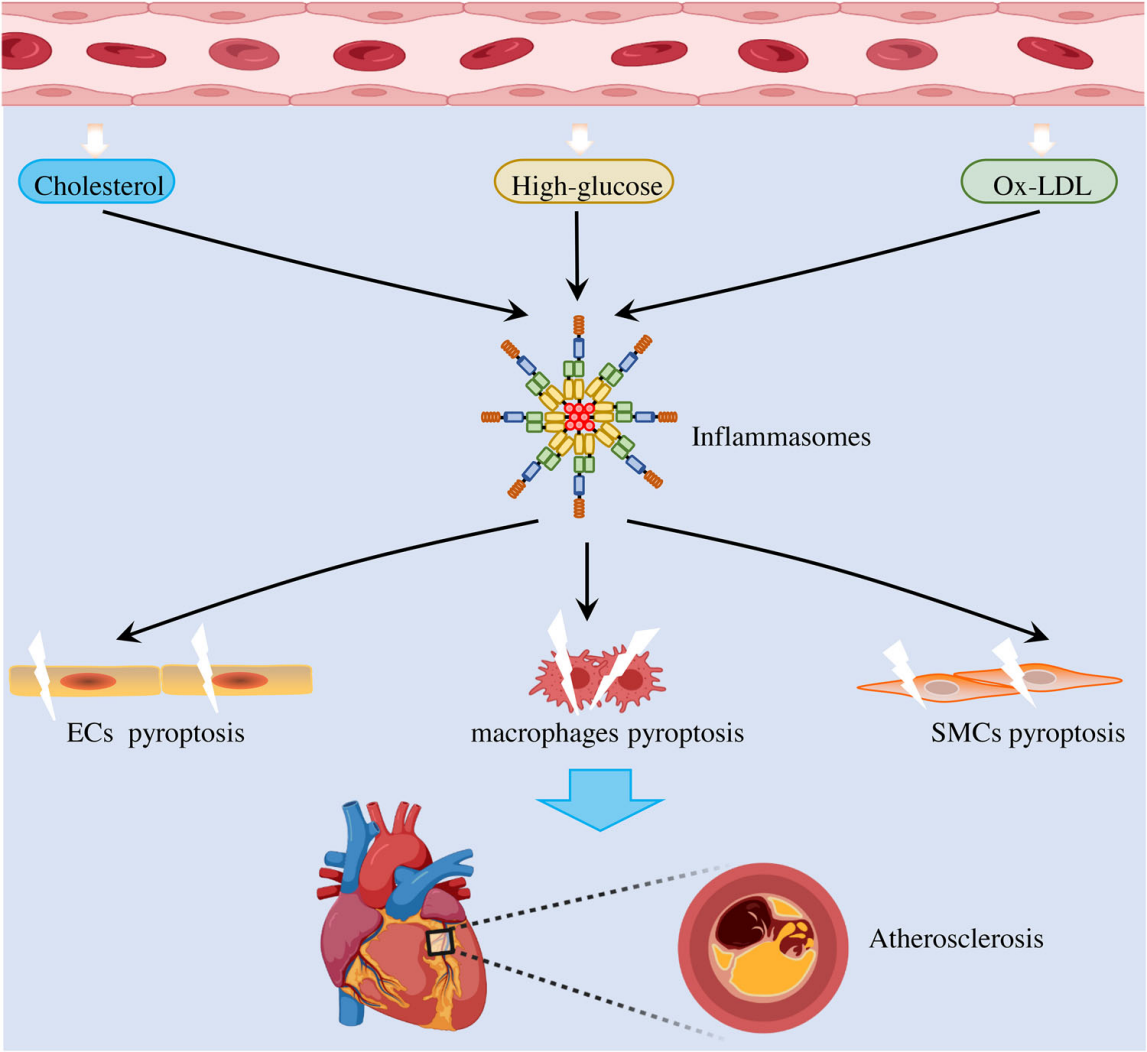
Multiple risk factors contribute to atherosclerosis by inducing inflammasome‐mediated pyroptosis. A large number of atherosclerotic‐related risk factors, such as cholesterol crystal, oxidized low‐density lipoprotein (ox‐LDL), and high‐glucose, can induce inflammasome activation. The subsequently activated inflammasome induces IL‐1β maturation and secretion to mediate inflammatory responses, as well as triggers endothelial cells (ECs), macrophages, and smooth muscle cells (SMCs) pyroptosis, both of which contribute to the pathogenesis of atherosclerosis.

Atherosclerosis is a well‐known CVD, which mainly involves abnormal lipid accumulation, immune cell infiltration, and proinflammatory cytokines activation in the aorta.^134^, ^135^, ^136^ Many risk factors are involved in the development of atherosclerosis, but the mechanisms are not completely understood. Recent studies have found that multiple risk factors trigger pyroptosis in atherosclerosis associated cells, including endothelial cells (ECs), macrophages, and smooth muscle cells (SMCs),^137^, ^138^, ^139^, ^140^ suggesting that pyroptosis plays an important role in the pathological development of atherosclerosis (Figure 3).

#### 3.1.1 ECs pyroptosis during atherosclerosis

ECs are essential for maintaining cardiovascular homeostasis, and its pyroptosis leads to the endothelium dysfunction and loss of integrity, thereby contributing to pathogenesis of atherosclerosis. Multiple atherosclerotic‐related risk factors have been found to cause ECs pyroptosis, such as cholesterol crystal, oxidized low‐density lipoprotein (ox‐LDL).^141^ Cholesterol crystals are a well‐known pivotal pathological marker of atherosclerotic plaque vulnerability; studies have shown that cholesterol crystal promotes atherosclerosis by inducing the activation of inflammasome and subsequent pyroptosis in ECs.^142^, ^143^, ^144^ Moreover, miR‐302c‐3p, a targeted negative regulator of NLRP3, blocks EC pyroptosis in a mouse model of atherosclerosis.^145^ ox‐LDL acts as an important initiator of atherosclerosis by promoting adhesion molecules expression and proinflammatory cytokines release. A recent study by Wu et al.^146^ confirmed that ox‐LDL can induce EC pyroptosis and inflammatory response. Mechanically, ox‐LDL upregulates mixed lineage kinase domain‐like (MLKL) expression in ECs, which augments NLRP3 inflammasome activation and pyroptosis.^146^ Notably, the inhibitor of NLRP3 inflammasome, MCC950, was able to block ox‐LDL‐induced pyroptosis, suggesting that MCC950 could be used as a promising treatment for atherosclerosis.^146^ Interestingly, MLKL is the terminal executor of necroptosis and is inextricably linked to pyroptosis.^118^ Therefore, it is necessary to distinguish the different roles of pyroptosis and necroptosis in ECs death during atherosclerosis. In addition to these endogenous metabolites, several exogenous substances have also been found to contribute to ECs pyroptosis and atherosclerosis. Nicotine is the main harmful ingredient of cigarette, which has been found to induce NLRP3 inflammasome activation and pyroptosis by facilitating reactive oxygen species (ROS) production in human aortic ECs (HAECs).^147^ Consistent with this finding, cadmium (Cd), another important and common environmental pollutant, has been implicated in atherosclerosis, but the mechanisms are not fully understood. Chen et al. discovered that treatment with Cd significantly increased NLRP3 inflammasome‐dependent pyroptosis by inducing mitochondrial ROS (mtROS) and intracellular ROS production in HUVECs. In general, ECs induce atherosclerosis through multiple downstream events upon pyroptosis occurs. On the one hand, ECs pyroptosis increase cardiovascular inflammation by inducing the mature and release of proinflammatory cytokines, such as IL‐1β and IL‐18, and subsequent recruiting monocytes to the endothelium for transformation into macrophages. On the other hand, ECs pyroptosis promote SMCs migration and deposition by causing endothelium dysfunction and increases permeability.

#### 3.1.2 Macrophages pyroptosis during atherosclerosis

Macrophages play an important role in the formation of plaques by engulfing the modified LDL to form foam cells, and their pyroptosis is involved in the pathogenesis of atherosclerosis.^148^ High‐glucose and ox‐LDL are major causes of atherosclerosis; a recent study found that treatment with glucose and ox‐LDL triggers macrophages pyroptosis in rats with diabetic atherosclerosis (DA).^149^ Similarly, Liu et al.^150^ found that ox‐LDL could also inhibit the cell viability by inducing pyroptosis in THP‐1‐derived macrophages. Moreover, blocking autophagy promotes macrophages pyroptosis through the p62/Nrf2/ARE pathway, which provides a promising therapeutic target for atherosclerosis.^150^ GSDMD as an executor of pyroptosis has recently been found to be involved in the pathogenesis of atherosclerosis.^151^ The expression of gasdermin D was upregulated in peripheral blood mononuclear cells (PBMCs) from patients with atherosclerosis. Moreover, GSDMD was activated in macrophages of ApoE^−/−^ mice on a high‐fat/high‐cholesterol (HFHC) diet, and blocking GSDMD in HFHC‐fed ApoE^−/−^ mice significantly reduced lesion volume and the number of infiltrated macrophages, suggesting that GSDMD can be act as a novel therapeutic target for atherosclerosis.^151^ In addition, nicotine also triggers macrophage pyroptosis in atherosclerotic lesions in an HDAC6‐dependent manner. In terms of the mechanism, HDAC6 mediates the acetylation of p65 and promotes NLRP3 transcription, targeting HDAC6 suppress nicotine‐induced pyroptosis in RAW264.7 cells.^152^ Notably, a recent study by Magupalli et al.^153^ found that HDAC6 can also promote NLRP3 inflammasome activation by inducing microtubule retrograde transport to microtubule‐organizing center, which is an important platform for inflammasome assembly. Overall, multiple risk factors can induce macrophages pyroptosis and then promote necrotic core formation and plaque instability in advanced lesions, which are continued to the pathogenesis of atherosclerosis.^154^

#### 3.1.3 SMCs pyroptosis during atherosclerosis

SMCs are the main stromal cells of the vascular wall, which are essential for maintaining cardiovascular homeostasis, and their abnormal function can lead to a variety of CVDs, including atherosclerosis. Studies have shown that SMCs pyroptosis leads to its dysfunction in response to various atherosclerotic‐related risk factors.^155^ Pan et al.^156^ found that high‐fat diet (HFD) promotes ICMA‐1 and GSDMD‐N expression and plaque lesion area by increasing AIM2 expression in ApoE^−/−^ mice. Moreover, in vitro studies showed that ox‐LDL accelerates GSDMD activity and SMCs pyroptosis through NF‐κB, AIM2, ASC, and caspase‐1 pathway in a concentration‐dependent manner.^156^ In parallel, a recent study by Liu et al.^157^ reported that LPS derived from *Porphyromonas gingivalis* (Pg‐LPS) could lead to SMCs pyroptosis depending on circRNA PPP1CC, and knockdown of circRNA PPP1CC relieved the expression of HMGB1, TLR9, and AIM2. Mechanically, circRNA PPP1CC directly targeted miR‐103a‐3p and miR‐107 to increase the expression of HMGB1, suggesting that circRNA PPP1CC may represent a novel therapeutic target for atherosclerosis by blocking SMCs pyroptosis.^157^ Anyway, SMCs are important for inhibiting plaque formation and maintaining plaque stability. Upon pyroptosis is induced by atherosclerotic‐related risk factors and leads to its dysfunction; the dysfunctional SMCs can release inflammatory cytokines, such as IL‐6, IL‐8, and other cytokines, to promote plaque formation in the early stages and also trigger an inflammatory response to destroy the fiber cap. Subsequently, the damaged fiber cap increases plaque instability and vulnerability, and ultimately leading to the development of atherosclerosis.^155^

### 3.2 Pyroptosis and myocardial infarction

MI is a common CVDs caused by prolonged ischemia of part of the myocardium upon the coronary artery thrombosis is occluded. Although the current treatment of MI has achieved rapid development and reperfusion can alleviate MI well, reperfusion will cause ischemia/reperfusion (I/R) injury (IRI) and aggravate fatal tissue damage. Recent studies have found that pyroptosis contributes to myocardium death and involves in the pathological process of MI.^158^, ^159^, ^160^ As previously mentioned, Mezzaroma et al. found that three components of the inflammasome, such as cryopyrin, ASC, and caspase‐1, were significantly increased in the granulation tissue and cardiomyocytes surrounding the infarct in an experimental mouse model of acute myocardial infarction (AMI). In addition, inflammasome formation was associated with increased cell death, myocardial infarction size, and cardiac enlargement after AMI. Moreover, inhibiting inflammasome formation by blocking purinergic receptor P2X, ligand gated ion channel, 7 (P2 × 7), an ATP gated ion channel has been found to promote NLRP3 inflammasome activation, and cryopyrin can limit cell death and alleviate MI, although the investigators did not determine whether pyroptosis was involved.^161^ Subsequently, Lei et al.^162^ reported that oxidative stress induced NLRP3 inflammasome‐mediated pyroptosis through the NF‐κB–GSDMD axis, which is contributed to cardiomyocytes loss following MI. Significantly, inhibited oxidative stress with N‐acetyl‐cysteine (NAC) or suppressed NF‐κB activation with pyrrolidine dithiocarbamate reduced GSDMD activation and pyroptosis, providing a promising target for MI‐related ventricular remodeling.^162^ Consistent with this finding, a recent study found that GSDMD activation and its mediated pyroptosis were upregulated in cardiomyocytes after IRI, and GSDMD deficiency in cardiomyocytes significantly reduced the myocardial infarct size induced by I/R, suggesting that GSDMD‐mediated cardiomyocyte pyroptosis exacerbates myocardial IRI.^163^ In addition to cardiomyocytes, inflammasome has also been found to be activated by inducing ROS production and potassium efflux in cardiac fibroblasts, another cell closely involved in MI development, to initiate the inflammatory response after myocardial IRI. Furthermore, in vivo studies also shown that inflammatory responses are inhibited in cardiac fibroblasts, but not in cardiomyocytes, from ASC or caspase‐1‐deficient mice and the subsequent myocardial dysfunction and infarction size are significantly alleviated.^164^ Although GSDMD‐mediated cardiomyocytes and cardiac fibroblasts pyroptosis plays an important role in the pathogenesis of MI, it is unclear whether the inflammatory response mediated by GSDMD is also involved in MI processes. Therefore, the detailed mechanism of pyroptosis in MI remains to be further explored.

### 3.3 Pyroptosis and hypertension

Hypertension, also known as elevated blood pressure, refers to the excessive force of blood hitting against the artery walls and is associated with a variety of CVDs. Recent studies have found that pyroptosis is closely related to the pathogenesis of hypertension.^134^, ^165^ It has been found that LPS and hyperhomocysteine (generally refers to the concentration of homocysteine in the serum above 10 μmol/L) are important risk factors for inducing hypertension by triggering EC damage.^166^, ^167^, ^168^ However, the mechanism of LPS and hyperhomocysteine‐induced EC dysfunction is not fully understood. Xi et al. found that homocysteine and/or LPS individually and synergistically induced aortic EC death by activating caspase‐1‐mediated pyroptosis in HUVEC during hypertension. Mechanically, homocysteine/LPS increases intracellular ROS levels, promotes NLRP3 inflammasome assembly and subsequent pyroptosis. Furthermore, treatment with caspase‐1 inhibitors or caspase‐1/NLRP3 deficiency could rescue hyperhomocysteine‐induced aortic EC dysfunction.^169^ In addition, it should be noted that homocysteine/LPS can also induce caspase‐3‐mediated‐EC apoptosis by promoting mitochondrial dysfunction and cytochrome *C* release. Interestingly, this process can be attenuated by antioxidants and caspase‐1 inhibitor, suggesting that apoptosis is a downstream event of caspase‐1 activation and ROS.^169^ Consistent with this finding, a recent study by Zhang et al. found that pyroptosis occurred in the media of pulmonary arteries in rat models of pulmonary hypertension (PH). Similarly, pyroptosis has also been found in hypoxic human pulmonary arterial SMCs (hPASMCs) under hypoxia in vitro. Furthermore, administered vx‐765 and ac‐YVAD‐CMK, two caspase‐1 inhibitors, suppressed pulmonary vascular fibrosis as well as alleviated the pathogenesis of PH by inhibiting SMCs pyroptosis.^170^ Interestingly, glioma‐associated oncogene family zinc finger 1 (GLI1), a transcriptional activator, was found to aggravate the pathological process of PH by promoting hypoxia‐induced PASMCs pyroptosis. Moreover, treatment with GLI1‐specific inhibitor GANT61 reduces PASMCs pyroptosis and alleviates PH during hypoxia. Mechanically, GLI1 enhances ASC expression by binding to its promoter, which promotes inflammasome activation and subsequent pyroptosis.^171^ These findings suggest that GLI1 is an important target for the molecular therapy of PH.

### 3.4 Pyroptosis and DCM

DCM refers to cardiac dysfunction in individuals with diabetes mellitus, including myocardial structure, functional and metabolic abnormalities in the absence of other risk factors, such as coronary artery disease (CAD) and hypertension.^172^, ^173^, ^174^ It is well known that cardiomyocyte death is the initiator of DCM, and recent studies have pointed out that pyroptosis plays an important role in the pathogenesis of DCM. Luo et al.^175^ demonstrated that NLRP3 inflammasome activation and pyroptosis were found in myocardium of diabetic rats, and silencing of NLRP3 in cardiomyocytes alleviates the pathological process of the DCM by suppressing cardiomyocyte pyroptosis under high glucose and. In addition, a recent study by Xie et al.^176^ discovered that chemerin can induce cardiomyocyte pyroptosis in G‐protein‐coupled chemokine‐like receptor 1 (CMKLR1) and NLRP3 inflammasome‐dependent manner. Furthermore, silencing of CMKLR1 with siRNA improves the function of cardiac in a Sprague–Dawley rat model of DCM induced by HFD and low dose of streptozotocin (STZ) by attenuating cardiac inflammation and cardiomyocyte pyroptosis, indicating that pyroptosis is an important protective target of DCM.^176^ Consistent, the bone morphogenetic protein‐7 facilitates cardiac repair and left ventricular heart function by attenuating TLR4–NLRP3 inflammasome axis‐induced pyroptosis in DCM.^173^ In addition, several studies have pointed out that noncoding RNA (ncRNA) also plays a critical role in regulating cardiomyocyte pyroptosis in DCM. Li et al.^177^ reported that the expression of mir‐30d was positively correlated with cardiomyocyte pyroptosis in STZ‐induced diabetic rats. Mechanistically, mir‐30d promotes caspase‐1 activation and cardiomyocyte pyroptosis through directly inhibiting the expression of foxo3a and its downstream transcription target protein, apoptosis repressor with caspase recruitment domain (ARC). Similarly, microRNA‐9 restrains hyperglycemia‐induced human hearts and ventricular cardiomyocyte pyroptosis by directly targeting ELAV‐like protein 1 in DCM.^178^ In addition, the long noncoding RNA (lncRNA) Kcnq1ot1 was found to competently regulate caspase‐1 expression with miR‐214‐3p. Knock downing Kcnq1ot1 by lentivirus‐shRNA or small interfering RNA improves cardiac function and fibrosis in DCM by inhibiting caspase‐1 expression and ameliorating cardiac fibroblasts pyroptosis.^179^ In conclusion, these results suggest that pyroptosis is an important pathogenic factor of DCM, and targeting pyroptosis is a promising approach for treating the disease. However, the function of GSDMD, a key executor of pyroptosis, in the pathogenesis of DCM is still unclear, and further investigation is needed.

### 3.5 Pyroptosis and other CVDs

In addition to the diseases mentioned above, pyroptosis has been found to be closely associated with a variety of other CVDs, such as dilated cardiomyopathy, arrhythmia, myocarditis, and cardiac hypertrophy.^141^, ^180^, ^181^, ^182^ Dilated cardiomyopathy is a common cause of heart failure. Zeng et al.^183^ provides evidence of cardiomyocyte pyroptosis in the heart. NLRP3 inflammasome activation through caspase‐1 will trigger cardiomyocyte pyroptosis to induce dilated cardiomyopathy, which will be regarded as a proper therapeutic target of dilated cardiomyopathy. Arrhythmia affects the life quality and threatens human life. Xu et al.^184^ reveal that aesculin could decrease the NLRP3 inflammasome activation and ameliorated the inflammatory response and NLRP3 inflammasome‐mediated pyroptosis of cardiomyocytes in neonatal rat cardiomyocytes and rats. Myocarditis is an inflammatory disease of the heart muscle. Liu et al. revealed that cholecalciterol cholesterol emulsion improves experimental autoimmune myocarditis in mice by downregulating the pyroptosis signaling pathway.^185^ In addition, the cysteine proteolytic enzyme cathepsin B has been reported to significantly exacerbate coxsackievirus B3 (CVB3)‐induced viral myocarditis by inducing inflammasome activation and its initiated myocardial pyroptosis.^186^ Moreover, inhibiting calpain with the endogenous inhibitor calpastatin improves CVB3‐induced viral myocarditis through inhibiting canonical or noncanonical pyroptosis pathways.^187^ Cardiac hypertrophy is initially as an adaptive response to physiological and pathological stimuli, which is the primary cause of mortality worldwide. Zhu et al. found that miR‐133a‐3p attenuates human myocardial cell line pyroptosis by directly targeting the 3′‐UTR of IKKε and suppressed its expression in angiotensin II‐induced cardiac hypertrophy. Moreover, NLRP3 inflammasome‐mediated pyroptosis contributes to cardiac hypertrophy induced by aortic constriction, and the inhibitor of NLRP3 inflammasome irisin can attenuate cardiac hypertrophy by inhibiting pyroptosis.^188^

### 3.6 Treatment of CVDs by targeting pyroptosis

Pyroptosis contributes to the development of CVDs, suggesting that inhibition of pyroptosis is a promising and effective strategy for the treatment of these diseases. Currently, numerous inhibitors of pyroptosis and its upstream inflammasome have been reported to alleviate CVDs^189^ (Table 3).

**TABLE 3:** Treatment of cardiovascular diseases by targeting pyroptosis.

Targets	Inhibitors	NLRP3 related diseases	References
NLRP3	MCC950	Atherosclerosis	^192^
		Myocardial infarction	^193^
	Melatonin	Atherosclerosis	^195^
		Myocardial infarction	^196^
	Colchicine	Atherosclerosis	^143^
	Sinapic acid	Diabetic atherosclerosis	^149^
	Hydroxytyrosol acetate	Atherosclerosis	^197^
	Tranilast	Coronary artery disease	^199^
		Myocardial fibrosis	^200^
	OLT1177	Myocardial infarction	^203^
	Oridonin	Myocardial infarction	^204^
Caspase‐1	VX‐765	Atherosclerosis	^208^
		Acute myocardial infarction	^158^
	z‐WEHD‐FMK	Diabetic cardiomyopathy	^209^
		Atherosclerosis	^210^
	Ac‐YVAD‐CMK	Ischemia/reperfusion injury	^211^
		Hypoxia/reoxygenation injury	^212^
	Ac‐YVAD‐CHO	Chronic kidney disease	^213^
GSDMD	Necrosulfonamide	Ischemia/reperfusion injury	^215^
	Disulfiram	Heart disease	^217^
	Dimethyl fumarate	Ischemia/reperfusion injury	^219^

#### 3.6.1 NLRP3 inhibitors

NLRP3 inflammasome activation is a key upstream event of pyroptosis, and its inhibition is an important strategy for preventing pyroptosis and alleviating CVDs.^190^ MCC950 is a small molecule inhibitor of NLRP3 that can alleviate a variety of inflammasome‐related diseases, including cryopyrin‐associated periodic syndrome (CAPS), experimental autoimmune encephalomyelitis (EAE), type 2 diabetes, and Alzheimer's disease (AD).^191^ Recent studies have found that MCC950 has a good protective effect on the development of atherosclerosis by alleviating macrophages pyroptosis and proinflammatory cytokine production in apoE^−/−^ mice fed with HFD.^192^ Moreover, MCC950 also reduces infarct size and protect the cardiac function from being weakened in a pig model of MI.^193^ It should be noted that although MCC950 has shown good efficacy in animal models of NLRP3 inflammasome‐related diseases, MCC950 failed in clinical trials due to safety issues.^194^ Therefore, the drug properties of MCC950 remain to be further explored. Melatonin (MT) is a hormone secreted by the brain pineal gland. Zhang et al. found that MT alleviates atherosclerosis via inhibiting EC pyroptosis in aortic intima of HFD‐fed ApoE^−/−^ mice. Mechanistically, treatment HAECs with MT upregulates the expression of lncRNA MEG3, an endogenous RNA that suppresses the function of miR‐223 by sequence complementarity, and promotes NLRP3 expression and inflammasome activation.^195^ In addition to alleviating the pathological process of atherosclerosis, MT has also been found to have cardioprotective effects via inhibiting NLRP3 inflammasome activation and its induced cardiomyocyte pyroptosis in mice with MI.^196^ Colchicine is an important plant extract that inhibits cell proliferation. A recent study shown that colchicine inhibits cholesterol crystal‐induced pyroptosis via triggering AMPK/SIRT1 pathway activation in human umbilical vein ECs (HUVECs).^143^ Furthermore, Magupalli et al.^153^ found that colchicine could also inhibit NLRP3 inflammasome activation by blocking microtubule polymerization.

In addition, sinapic acid was found to suppress macrophages pyroptosis in DA, and treatment with low‐dose (≤50 mg/kg) sinapic acid inhibits the levels of endothelin 1 (ET‐1) and proinflammatory cytokine IL‐1β in serum by downregulating the expression of lncRNA‐metastasis‐associated lung adenocarcinoma transcript 1 (MALAT1).^149^ Hydroxytyrosol acetate (HT‐AC), a natural polyphenolic compound derived from olive oil, has recently been found to inhibit vascular EC pyroptosis and atherosclerotic lesions formation via targeting the downregulated expression of HDAC11 in HFD‐fed ApoE^−/−^ mice.^197^ However, the mechanism of HDAC11 regulating pyroptosis is still unclear and needs to be further explored. In addition to the inhibitors mentioned above, several other drugs and molecules that inhibit inflammasome activation have been found to alleviate CVDs. Tranilast is a targeted inhibitor of inflammasome by directly binding to NLRP3^198^ and has been shown to be effective in the treatment of CAD and myocardial fibrosis.^199^, ^200^ Similarly, OLT1177 and oridonin also interact directly with NLRP3 and inhibit inflammasome activation,^201^, ^202^ reducing infarct size and relieving MI after IRI in the mouse.^203^, ^204^ Moreover, glyburide and 3,4‐methylenedioxy‐β‐nitrostyrene are two other small molecule compounds that have been found to inhibit inflammasome activation,^205^, ^206^ but their protective effect on CVD is unclear. Together, these studies suggest that indirect targeting of pyroptosis by inhibiting inflammasome can treat CVDs, but the important role of pyroptosis in this process needs to be further clarified.

#### 3.6.2 Caspase‐1 inhibitors

Similar to NLRP3, caspase‐1 is another key molecule in the initiation of pyroptosis by direct cleaving of GSDMD, and its inhibitors have also been found for the treatment of CVDs, such as VX‐765, z‐WEHD‐FMK, ac‐YVAD‐CMK, and Ac‐YVAD‐CHO.^207^ VX‐765 is a specific inhibitor of caspase‐1, which has been found to attenuate the development and progression of atherosclerosis in ApoE‐deficient mice by inhibiting VSMCs pyroptosis.^208^ In addition, VX‐765 treatment also significantly reduced the infarct size and cardiomyocyte pyroptosis in the mouse model of AMI.^158^ Benzyloxycarbonyl‐Trp‐Glu(OMe)‐His‐Asp(Ome)‐fluoromethylketone (z‐WEHD‐FMK) is an irreversible inhibitor of caspase‐1 that inhibits vascular neointima hyperplasia by preventing VSMCs proliferation and migration in diabetic mice.^209^ Furthermore, Wang et al.^210^ reported that treatment with z‐WEHD‐FMK alleviates hyperhomocysteinemia‐induced atherosclerosis in apoE‐deficient mice. Ac‐YVAD‐CMK is a tetrapeptide sequence that irreversible inhibits caspase‐1 by targeting the sequence of caspase‐1 in pro‑IL‑1β, treatment with Ac‐YVAD‐CMK significantly restrains hypoxia‐reoxygenation‐induced troponin I (TnI), an effective diagnostic biomarker for myocardial infarction, degradation by inhibiting MMP‐2 activity in a dose‐dependent manner in neonatal cardiomyocytes.^211^ Furthermore, Ac‐YVAD‐CMK attenuates high glucose (HG)‐ and hypoxia/reoxygenation (H/R)‐induced H9C2 cell injury by blocking NLRP3 inflammasome‐mediated pyroptosis.^212^ Similar to Ac‐YVAD‐CMK, Ac‐YVAD‐CHO is also a specific tetrapeptide inhibitor of caspase‐1, with IC_50_ values of 2.5 μM in mice. It has been found that Ac‐YVAD‐CHO can partially reverse uric acid‐induced vascular endothelial injury and play a protective role in the cardiovascular system.^213^

#### 3.6.3 GSDMD inhibitors

GSDMD is the main executor of pyroptosis, unlike caspase‐1 and NLRP3 inflammasome inhibitors, the inhibitors of GSDMD have been less reported. Necrosulfonamide (NSA) was first found to inhibit necroptotic by directly binding to MLKL, but a recent study by Rathkey et al. demonstrated that NSA can directly interact with GSDMD via Cys191 and thus act as a selective inhibitor of GSDMD.^214^ In the rat model of pulmonary IRI, administration of NSA significantly improves the physiological functions of lung.^215^ However, this study suggests that the protective effect of NSA on IRI depends on its inhibitory effect on necroptosis, and the role of pyroptosis in IRI remains to be further explored. Consistent with NSA, disulfiram (DSF), an old drug that has been approved for treating alcohol addiction in 1951 by the United States Food and Drug Administration, was also found to inhibit pore formation and pyroptosis by targeting human GSDMD at Cys191.^216^ Although the protective role of DSF in heart disease has been mentioned,^217^ its effect and mechanism remain to be elucidated in detail. In addition, exogenously dimethyl fumarate (DMF) and endogenous fumarate block the interaction between GSDMD and caspase‐1, and then inhibit pyroptosis by inducing GSDMD succination at Cys191.^218^ Interestingly, DMF has been reported to protect cardiomyocytes from injury in an oxygen‐glucose deprivation/reoxygenation model of myocardial IRI. But the therapeutic effect of DMF on AMI depends on its inhibitory effect on apoptosis rather than pyroptosis.^219^ Notably, a recent study shown that the ragulator–rag–mTORC1 pathway is required for GSDMD pore formation and pyroptosis rather than GSDMD cleavage. Mechanistically, mTORC1 promotes GSDMD oligomerization by inducing ROS production and mitochondria damage.^220^ These results show that the Ragulator–Rag–mTORC1 complex is a necessary regulator for GSDMD oligomerization, indicating that targeting this pathway may improve CVD by suppressing pyroptosis.

## 4 PYROPTOSIS AND CANCER

At present, malignant tumors have become one of the most serious diseases endangering human health.^221^, ^222^, ^223^ The tumor occurrence and development, which need detailed research to search proper treatments to improve the survival rate of patients, are affected by a variety of factors, including oncogene activity, oxidative stress, immune microenvironment, and chronic inflammation.^224^, ^225^, ^226^ Particularly, pyroptosis induces inflammatory cytokines release, such as IL‐1 and IL‐18, which could induce an inflammatory environment to increase tumor infiltration and promote the likelihood of tumorigenesis and metastasis.^227^, ^228^, ^229^ Meanwhile, pyroptosis occurs in almost all the type of cancer and shows a double‐edged sword effect to cancers, which could either enhance or restrain tumorigenesis.^230^, ^231^, ^232^, ^233^ Therefore, we need to deeply explore the specific mechanism of pyroptosis and tumor progression to provide evidence for tumor prevention and treatment (Table 4).

**TABLE 4:** The role of pyroptosis in different diseases

Disease	Pyroptosis functions	References
Breast cancer	miR‐1290/NLRP3‐mediated decreases the tumor radioresistance	^235^
	GSDME‐mediated pyroptosis inhibits the tumor growth	^236^, ^237^, ^238^, ^239^, ^240^, ^241^, ^242^
	GSDMD‐mediated pyroptosis suppresses the tumor growth	^243^, ^244^, ^245^
Lung cancer	GSDMD‐dependent pyroptosis enhances antitumor therapy	^249^, ^250^, ^251^, ^252^, ^253^, ^254^
Colorectal cancer	GSDME‐dependent pyroptosis inhibits the tumor progression	^256^, ^257^, ^258^, ^259^, ^260^
Gastric cancer	NLRP3‐dependent pyroptosis promotes cisplatin sensitivity for cancer therapy	^261^
	Enhanced GSDMD cleavage could achieve cell pyroptosis to inhibit tumor growth	^264^, ^265^
	GSDME‐mediated pyroptosis enhances antitumor effect	^266^, ^267^
Liver cancer	GSDME‐dependent pyroptosis promotes cancer therapy	^268^, ^269^
	Macrophages pyroptosis enhances NK‐cell response for immunotherapy	^270^
	NLRP3 inflammasome‐dependent pyroptosis promotes tumor suppression	^271^
Parkinson's disease	Inhibition of inflammasome activation and pyroptosis can prevent dopaminergic neuron death	^286^, ^287^
	Inhibition of pyroptosis can improve behavioral disorders and reduce nigrostriatal dopaminergic degeneration in MPTP mouse model	^291^, ^292^
Alzheimer disease	Inhibition of caspase‐1 alleviates neuronal injury and neuroinflammation	^301^
Stroke	Caspase‐1‐mediated pyroptosis leads to neuroinflammation	^311^
	Caspase‐1‐mediated pyroptosis leads to neuronal death and cerebrovascular destruction	^312^
Amyotrophic lateral sclerosis	NLRP3‐mediated pyroptosis leads to neuronal death and motor neuron degeneration	^323^
Diabetes	NLRP3‐mediated pyroptosis impairs the regulation of glucose homeostasis and metabolism	^314^, ^329^
	NLRP3‐mediated pyroptosis leads to impaired islet β cell function	^337^
Obesity	Caspase‐11‐mediated pyroptosis leads to degeneration of colonic intermuscular nitrogenergic neurons and colonic dyskinesia	^340^
	Caspase‐1/4/5 activates pyroptosis leading inflammatory response in adipose tissue	^345^, ^346^
Gout	NlLRP3‐dependent pyroptosis leads inflammatory response in the joints	^348^

### 4.1 Pyroptosis and breast cancer

In recent years, the new cases of breast cancer have increased dramatically, becoming the largest cancer in the world.^234^ Research into effective breast cancer prevention and treatment strategies remains a huge challenge. The expression of miR‐1290 shows higher level in radioresistant tumor tissues of triple‐negative breast cancer (TNBC) patients, which could inhibit the radiation‐induced pyroptosis with human breast cancer cells (MDA‐MB‐231) radiosensitivity reduced. The potential target of miR‐1290 was NLRP3, and miR‐1290/NLRP3‐mediated pyroptosis has a good potential to decrease the radioresistance in TNBC to be served as a novel antitumor strategy.^235^ GSDME is the core of pyroptosis and plays a significant role in breast cancer cells, which has a great potential to suppress the tumor growth and metastasis. Enhanced mitochondrial ROS could induce caspase‐3‐dependent cleavage of GSDME to promote pyroptotic cell death for inhibiting metastasis and proliferation of human breast cancer cells (MDA‐MB‐231).^236^ Triclabendazole could induce GSDME‐dependent pyroptosis through caspase‐3 activation, which shows a good potential for breast cancer therapy.^237^ Acute Cd exposure induces caspase 3‐GSDME‐mediated pyroptosis to suppress tumor growth of MDA‐MB‐231 breast cancer cells by NLRP3 inflammasome activation and ROS generation.^238^ Tumor suppressor DRD2 could induce GSDME‐mediated murine 4T1 breast cancer cells pyroptosis and educate macrophage to enhance antitumor efficacy.^239^ Mitochondrial uncoupling protein 1 could activate mitophagy and pyroptosis to inhibit the process of TNBC.^240^ Because of the high content of glutathione in murine 4T1 breast tumor microenvironment, the combination of chlorin e6 (Ce6) and heat shock protein 90 inhibitor tanespimycin (17‐AAG) could induce GSDME‐mediated pyroptosis and decrease myeloid‐derived suppressor cells, which could sensitize tumors of antiprogrammed death‐1 (PD‐1) therapy and enforce immunogenic photodynamic‐immune therapy.^241^ NI‐TA is a photocatalytic superoxide radical generator, which could trigger pyroptosis in MDA‐MB‐231 breast cancer via a caspase‐3/GSDME pathway for excellent stemness inhibition and tumor growth suppression.^242^ Meanwhile, GSDMD is also a significant target in breast cancer therapy. Cisplatin upregulates the lncRNA maternally expressed gene 3 to activate NLRP3/caspase‐1/GSDMD pyroptosis pathway for TNBC patients’ therapeutic enhancement.^243^ A bacterium‐attenuated *S. typhimurium* (VNP) system is developed to deliver GSDMD into murine 4T1 tumor cells to initiate GSDMD‐triggered pyroptosis for immunotherapy. The strategy based on tumor pyroptosis has a great chance to enhance adoptive T‐cell therapy and cancer vaccines.^244^ Niu et al. construct a drug delivery system including nigericin (Nig) and decitabine (DAC). Nig could activate NLRP3 inflammasome and caspase‐1 protein to cleave GSDMD regulated by DAC, which could trigger murine 4T1 tumor cell pyroptosis for systemic anticancer immunity.^245^

### 4.2 Pyroptosis and lung cancer

Lung cancer is one of the most common malignant tumors, which has a high morbidity and mortality rate around the world with a significant threat to human health.^246^ GSDMD is a crucial factor of pyroptosis and overexpressed in non‐small cell lung cancer (NSCLC) cells, which is closely related to the larger tumor size and lymph nodes metastasis.^247^, ^248^ Ophiopogonin B (OP‐B) is a bioactive component from *Radix Ophiopogon Japonicus*, which shows high cell proliferation inhibition of NSCLC cells. It is verified that OP‐B induce caspase‐1/GSDMD‐dependent pyroptosis to reverse cisplatin resistant A549 cells.^249^ Trichosanthin could increase the expression of pyroptosis‐related proteins, such as GSDMD and NLRP3, to induce the pyroptosis of A549 NSCLC.^250^ Cucurbitacin B (CuB), a bioactive component from muskmelon pedicel, could bound the TLR4 to activate the NLRP3 inflammasome and separate of N‐ and C‐terminals of GSDMD to induce TLR4/NLRP3/GSDMD‐dependent pyroptosis for antitumor therapy of A549 NSCLC.^251^ Polyphyllin VI (PPVI), a bioactive component from *Trillium tschonoskii* Maxim, could increase ROS level to activate NF‐κB signaling pathway and NLRP3 inflammasome in A549 cells. The study demonstrates PPVI‐triggered caspase‐1‐mediated pyroptosis was closely related with ROS/NF‐κB/NLRP3/GSDMD signal axis in NSCLC.^252^ A combination system of a ruthenium (II) polypyridyl complex and Taxol could activate the pyroptosis key molecules of caspase‐1 and GSDMD to trigger caspase‐1/GSDMD‐mediated pyroptosis of A549 tumor cells for enhanced anticancer therapeutic effect.^253^ Ning et al. demonstrate that mixed‐lineage leukemia 4 ablation decreases the expression of DNA methytransferases and RNA‐induced silencing complex, which causes GSDMD‐dependent pyroptosis and transcriptional reactivation of double‐stranded RNA for strengthened immunotherapy in human lung cancer (H1299) cells. It reveals a general function of tumor‐cell GSDMD‐induced pyroptosis in enhancing anticancer immunity.^254^

### 4.3 Pyroptosis and CRC

CRC is one of the most common tumors worldwide, which seriously threatens human life and health.^255^ A synthetic farnesoid X receptor agonist (GW4064) could induce BAX/caspase‐3/GSDME‐mediated pyroptosis to promote the efficacy of oxaliplatin so as for enhanced anticancer effects of human CRC.^256^ In colitis‐associated CRC, released HMGB1 could trigger GSDME‐mediated pyroptosis to promote tumor cells proliferation by ERK1/2 pathway. How to inhibit GSDME‐mediated pyroptosis is one of a promising target for colitis‐associated human CRC therapeutic strategies.^257^ Gambogic acid (GA) could regulate the activation of caspase‐3 and induce the GSDME‐dependent pyroptosis to inhibit human CRC cells proliferation. Meanwhile, GA‐induced pyroptosis promotes proportions of DCs and CTLs in tumor microenvironment to enhance antitumor immune response.^258^ Apoptin from the VP3 gene of chicken anemia virus can increase the intracellular ROS and cleave caspase‐3 to trigger pyroptosis with GSDME cleavage. Apoptin induces HCT116 cells pyroptosis via the mitochondrial GSDME‐mediated apoptotic pathway for CRC therapy.^32^ The expression of GSDME could sensitize radio‐resistant tumor cells to radiation in human CRC. The radiation‐induced CRC cells pyroptosis via caspase‐3‐mediated pathway is determined by GSDME, which activates NK cells to promote anticancer immunity.^259^ The lobaplatin increase ROS expression and JNK phosphorylation to recruit Bax to mithochondria, thereby trigger pyroptosis to cleave GSDME due to the activation of caspase‐3. The mechanism of lobaplation against human CRC cancer is related to GSDME‐dependent pyroptosis, which may have promising in the anticancer clinical application.^260^

### 4.4 Pyroptosis and GC

GC has a high degree of malignancy and high rate of recurrence and metastasis in advance stage. Low‐dose diosbulbin‐B (DB) could inhibit properties of cancer stem cells and induce PD‐L1 depletion to activate NLRP3‐dependent pyroptosis, which effetely sensitize cisplatin‐resistant GC cells. Low‐dose DB induces PD‐L1/NLRP3 pathway pyroptosis to promote sensitivity of cisplatin in GC, indicating an appropriate strategy of GC treatment.^261^ As reported, GSDMA and GSDMC are decreased in GC compared with normal gastric tissue and may be regarded as antioncogene.^262^, ^263^ GSDMD is silenced in GC compared to adjacent normal tissues and decreased expression could trigger proliferation of cancer cells.^264^ A system of fructose‐coated Ångstrom‐scale silver particles (F‐AgÅPs) is established. F‐AgÅPs could induce LDH release, caspase‐1 expression, and GSDMD cleavage to achieve BGC‐823 cells pyroptosis in vitro and in vivo, indicating a promising therapeutic treatment for GC.^265^ Additionally, a combination of BIX‐01294 (BIX) and cisplatin shows human cancer cell pyroptosis with cleavage of GSDME and caspase‐3. The study first to verify BIX could induce GSDME‐mediated pyroptosis by autophagic flux activated to enhance antitumor effect.^266^ Cold atmospheric plasma (CAP) could effectively trigger GC pyroptosis depended on the cleavage of GSDME and the activation of mitochondrial pathways, which provides a novel strategy for anticancer treatment.^267^

### 4.5 Pyroptosis and liver cancer

HCC accounts for the majority of primary liver cancers and shows a serious threat to human health. Miltirone is a bioactivate molecule isolated from the root of *Salvia miltiorrhiza* Bunge. Miltirone could elicit ROS generation, inhibit the regulated ERK1/2 extracellular and MEK phosphorylation for pyroptosis induction with cleavage of GSDME and caspase‐3. Caspase‐3 siRNA‐mediated silencing attenuates the induction of miltirone on GSDME‐mediated human HCC pyroptosis.^268^ As_2_O_3_ nanoparticles (As_2_O_3_‐NPs) could promote more LDH release and trigger pyroptosis in GSDME‐expressing human HCC cancer cells. As_2_O_3_‐NPs activates caspase‐3 to cleave GSDME with free N‐terminal domain releasing, indicating emergence of pyroptosis.^269^ Sorafenib is an inhibitor of multitarget kinase, which is used to treat HCC in clinical. Sorafenib could induce macrophages pyroptosis, which approves direct immune modulation and decreases major histocompatibility complex class I expression for favor NK‐cell response, indicating a promising immunotherapy.^270^ Alpinumisoflavone, an anticancer drug for the treatment of HCC, could effectively induce cancer cell pyroptosis through NLRP3 inflammasome‐dependent pathway to increase the expression of pyroptosis‐related genes for enhanced tumor inhibition.^271^

### 4.6 Prospects of anticancer therapy by targeting pyroptosis

**FIGURE 4:**
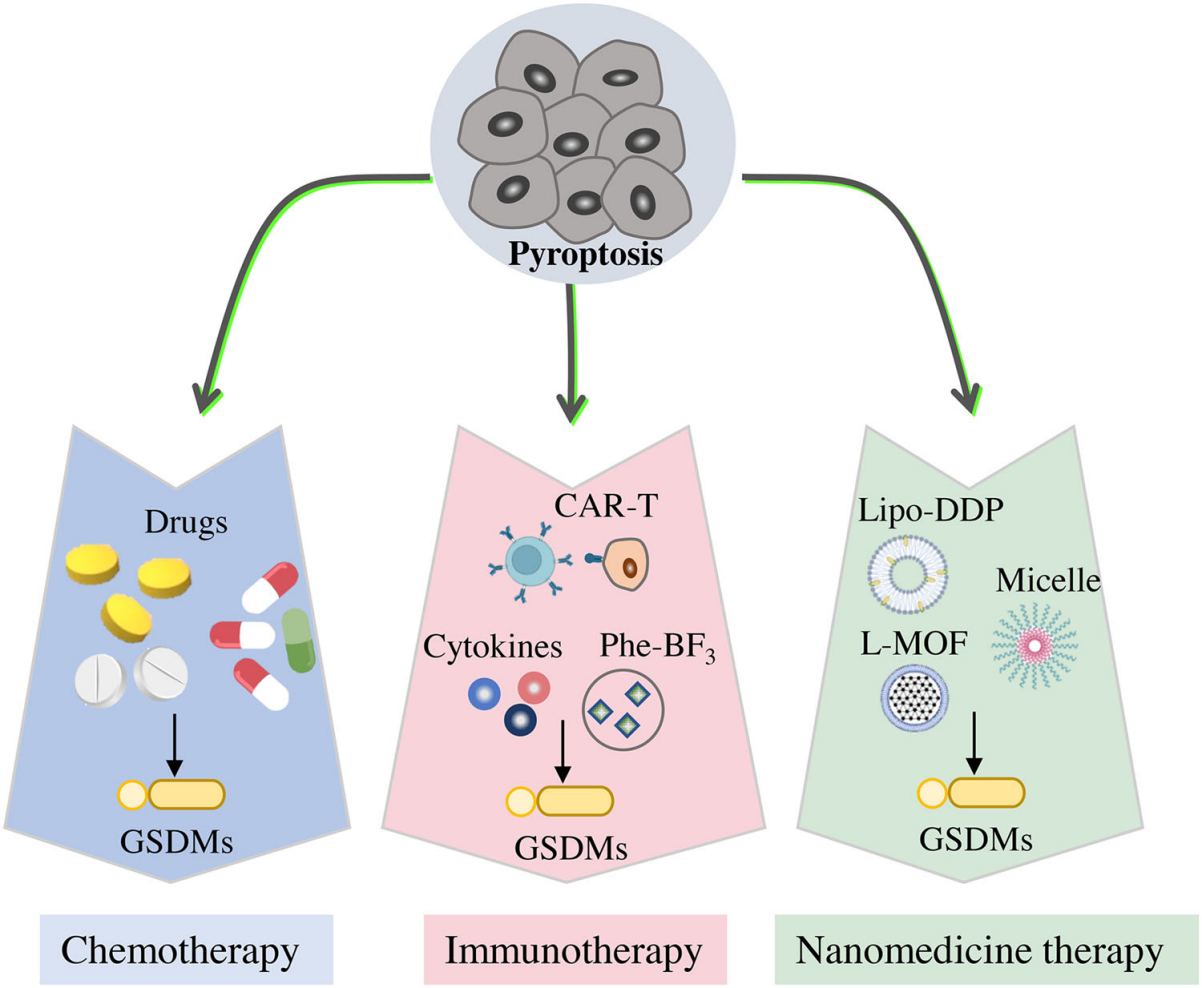
Prospects of anticancer therapy by targeting pyroptosis. The feasibility and potential of targeting pyroptosis as an antitumor therapy is investigated in recent years. Chemotherapy remains the most common cancer treatment, as it induces pyroptosis of tumor cells to cause cell death. Furthermore, some distinctive strategies have been investigated, including immunotherapy and nanomedicine therapy, all of which can effectively trigger pyroptosis in tumor cells.

Pyroptosis plays an important role in tumor occurrence and development, which is related to both tumor‐suppressing and tumor‐promoting effects. On the one hand, the adverse tumor environment in cancer cells induces long‐term chronic pyroptosis, which could enhance cancer progression. The proinflammatory cytokines induced by chronic pyroptosis promote the formation and maintenance of inflammatory environment for tumor progression. On the other hand, the activation of pyroptosis in cancer cells leads to infiltration of immune cells, which could activate the tumor immune response to inhibit the tumor growth (Figure 4).

#### 4.6.1 Chemotherapeutic drugs

As reported, chemotherapy is demonstrated it could trigger pyroptosis to induce cancer cell death, thus inhibits tumor growth. The pyroptosis of epithelial ovarian cancer cells could be triggered by 2‐(anaphthoyl) ethyl‐trimethylammonium iodide (α‐NETA) through pathway of caspase‐4/GSDMD.^272^ α‐NETA exhibits much lower cytotoxic effect after the knockdown of either caspase‐4 or GSDMD in ovarian cancer cells and decreases the growth of epithelial ovarian tumor in vivo. The results indicate that a‐NETA is a promising molecule of antitumor for cancer therapy by pyroptosis. Metformin could target miR‐497/leucine‐rich protein (PELP1) axis to promote pyroptosis of esophageal squamous cell carcinoma by GSDMD pathway.^273^ DOX could silence the expression of eukaryotic elongation factor‐2 kinase (eEF‐2K), which plays an important role in human melanoma cells through DOX‐induced pyroptosis, to promote the sensitivity of cancer cells to DOX.^274^ Lobaplatin increases the expression of ROS and JNK phosphorylation to induce GSDME‐dependent pyroptosis and caspase‐3/9 cleavage, thereby promoting the suppression of colon tumor growth.^260^ Cisplatin can trigger caspase‐3 and GSDME‐dependent pyroptosis in A549 cells, indicating cisplatin has a good potential in lung cancer therapy with high expression of GSDME.^275^ The pyroptosis in GC cells is induced by 5‐fluorouracil, accompanied by accumulation of the GSDME N‐terminal segment and generation of cleaved caspase‐3.^276^ Anthocyanidins could increase the expression of caspase‐1, NLRP3, and IL‐1β to induce pyroptosis, which enhances anticancer effect of oral squamous cell carcinoma cells.^277^

#### 4.6.2 Cancer immune stimulation by pyroptosis

The association of pyroptosis and anticancer immunity is tight. Granzyme B secreted by NK cells cleaves GSDME directly or activates caspase‐3 to cleave GSDME indirectly, inducing the pyroptosis of GSDME‐expressing cancer cells. The experiments in vivo demonstrated that tumor growth is not suppressed in nude mice, indicating that tumor growth inhibition through pyroptosis is related with the host immune system.^278^ A biorthogonal system of gold nanoparticles and phenylalanine trifluoroborate could deliver GSDMA3 into tumor cells to induce pyroptosis, thereby increasing the populations of NK cells and cytotoxic T cells. The pyroptotic tumor cells trigger anticancer immune response for enhanced PD‐L1 combined therapy.^279^ The tumor cells pyroptosis is triggered by NK cells and CD8^+^ T cells through granzyme‐A/GSDMB axis. The experiments reveal that the GSDMB triggers lytic death of target cell by granzyme A in NK cells.^280^ The released granzyme B and perforin trigger caspase‐3/GSDME‐induced cancer cell pyroptosis to achieve CAR‐T‐cell cancer therapy. The cancer cell pyroptosis also leads to the activation of capase‐1/GSDMD pathway in macrophages with proinflammatory cytokines release, triggering cytokine release syndrome.^281^ In hypoxic tumor cells, PD‐L1 nuclear translocation increases the production of GSDMC, then converts apoptosis to pyroptosis. The mechanism of nuclear PD‐L1 in hypoxia‐dependent pyroptosis is investigated, which proves the complex of PD‐L1/p‐Y705‐Stat3 induces pyroptosis through caspase‐8/GSDMC pathway.^76^

#### 4.6.3 Cancer pyroptosis and nanomedicine

Up to now, the chemical drug combinations have shown splendid efficacy in clinic, whereas the challenges of low solubility and nontargeted properties of drugs are remained. Because of rapid development of nanotechnology, it has a great opportunity to be a strategy to reduce the side effects and promote bioavailability of drugs.^282^ How to combine tumor cell pyroptosis and nanotechnology to suppress cancer progression has become a research hotspot. Lipo‐DDP, cisplatin loaded in liposome, could induce tumor cell pyroptosis by caspase‐3‐depedented pathway. The combination of Lipo‐DDP and DAC (a DNA methyl‐transferase inhibitor) triggers system immune response to suppress the tumor growth and metastasis.^283^ To promote the tumor accumulation of As_2_O_3_, a triblock polymer of mPEG–PLGA–PLL is prepared to construct nanomedicine with As_2_O_3_ enveloped. The As_2_O_3_ loaded in nanomedicine could increase GSDME‐N expression and trigger pyroptotic cell death.^269^ Metal‐organic framework (MOF) nanoparticles are regarded as promising carriers to deliver drugs into tumor. The lipid‐coated MIL‐100 (L‐MOF) is designed to deliver amounts Fe^3+^ ions into cancer cells. Large amounts of Fe^3+^ induce lysosomal rupture and pyroptosis.^284^ The nanocarrier with controlled release is a promising strategy to eliminate cancer cells by pyroptosis.

## 5 PYROPTOSIS AND NDs

NDs manifested as dysfunction or loss of nervous system function. Neuroinflammation driven by NLRP3 inflammasome in NDs has been proved, such as AD, Parkinson's disease (PD), stroke, and amyotrophic lateral sclerosis (ALS). Next, we will introduce the involvement of pyroptosis in NDs specifically (Table 4).

### 5.1 Pyroptosis and PD

PD is a common neurodegenerative disease, which is mainly due to the degeneration and death of dopaminergic neurons in the substantia nigra of midbrain. The development of PD is accompanied by the loss and appearance of dopaminergic neurons.^285^ Activation of the inflammasome has also been implicated in PD, recent studies have shown that suppression of the inflammasome and prevents dopaminergic neuron death in a 1‐methyl‐4‐phenyl‐1,2,3,6‐tetrahydropyridine (MPTP)‐induced PD mouse model.^286^, ^287^ In an MPTP‐induced PD mouse model, the release of IL‐1β was induced significantly higher than in controls, IL‐1β levels can be inhibited by MCC950.^288^, ^289^ In microglia, α‐synuclein aggregates into Lewy bodies, and α‐synuclein aggregates activate NLRP3 to promote the release of proinflammatory cytokines such as IL‐1β.^290^ In addition, inhibiting the occurrence of pyroptosis can improve behavioral disorders, reduce nigrostriatal dopaminergic degeneration, neuroinflammation, and inhibit the activation of proinflammatory microglia of PD rats.^291^, ^292^ Moreover, Anderson et al.^293^ findings suggest that midbrain inflammasome protein expression is a histopathological marker of early substantia nigra degeneration in PD patients. Li et al. found that high expression of microRNA‐188‐3p inhibit pyroptosis by targeting NLRP3 in MPTP‐induced PD mice and MN9D cells.^294^ At present, the clinical treatment of PD patients is mainly to increase the content of dopamine transmitters in the brain. However, considering that neuroinflammation has been shown to be an important pathogenesis of PD, alleviating the damage of dopamine neurons caused by inflammatory factors is also crucial for PD treatment.^295^, ^296^

### 5.2 Pyroptosis and AD

Many evidences support a significant role for inflammasomes in the pathogenesis of AD.^297^ IL‐18 was expressed in microglia and astrocytes, microglia‐derived proinflammatory cytokines are thought to be involved in AD and IL‐1β and IL‐18 aggravate the disease.^298^, ^299^ The shuttle of Amyloid β‐protein (Aβ) into mitochondria promotes the activation of DRP1 and aggravates mitochondrial dysfunction in the hippocampus of 5xFAD mice, which in turn induces the activation of NLRP3 inflammasome, leading to IL‐1β secretion and pyroptosis‐related protein GSDMD activation.^300^ Knockdown of NLRP1 or caspase‐1 reduced neuronal damage in an amyloid mouse model of AD. However, inhibition of caspase‐1 also blocks the release of inflammatory factors, so it is unclear whether neuronal damage is induced by inflammation or pyroptosis. In vitro, oligomeric fibers of Aβ induced ASC and NLRP3‐dependent IL‐1β release in astrocyte and microglia.^301^ Extracellular ASC have been shown to contribute to disseminate of amyloid and aggregate AD. Brain lysates bound to fibrous ASC spots were detected in animal models of AD pathology. This suggests that ASC plays a role in planting Aβ plaques.^302^, ^303^ What is more, both NLRP3 and caspase‐1 knockout mice showed improved memory compared with wild‐type mice.^304^ Recent studies have shown that targeting amyloid alone may not be sufficient to prevent AD and exploring the treatment of AD by targeting inflammasome pyroptosis is an important research avenue. Microglia‐derived proinflammatory cytokines IL‐1β and IL‐18 are thought to exacerbate AD.^305^ IL‐18 and IL‐1β were detected in microglia and neurons in the brains of AD patients, and IL‐18 showed colocalization with Aβ plaques and Tau protein.^306^ In AD models, the NLRP3 inhibitor mefenamic acid was shown to have no effect on pyroptosis, but inhibition or caspase‐1 deficiency protected neuroinflammation and memory deficits in rat models of Aβ‐induced AD.^307^ Tian et al.^308^ findings elucidate the crucial mechanisms of NLRP3/caspase‐1 in pyroptosis and tau pathogenesis induced by sevoflurane. Salidroside (Sal) can not only reduce and inhibit pyroptosis through accumulation of Aβ and phosphorylation of Tau through downregulation of IL‐1β and IL‐18 expression, in addition, Sal reversed the increase in the protein expression of TLR4, NF‐κB, NLRP3, ASC, cleaved caspase‐1, cleaved GSDMD, IL‐1β, and IL‐18 in AD mouse model.^309^ Targeting amyloid alone may not be enough to prevent AD, exploring the drivers of the inflammasome in AD pathology is an important avenue of research.

### 5.3 Pyroptosis and stroke

Stroke‐induced neuroinflammation can cause delayed neuronal death. Other studies have shown that inflammasome can be activated for pyroptosis in the brain after ischemic stroke. Neuronal caspase‐1 may be activated early in infarction, and in vivo, the activation of caspase‐1 was inhibited using intraventricular Ac‐YVAD‐cmk.^310^ IL‐1β is one of the major proinflammatory cytokines mediating neuroinflammation, which acts by amplifying neuroinflammation, leading to microglia‐mediated neuronal death or vascular destruction, and inhibition or knockdown of caspase‐1 has neuroprotective effects in focal stroke models.^311^ Cerebral ischemia can initiate the inflammatory response of microglia and promote the formation of inflammasomes, such as NLRP1, NLRP3, and NLRP4 proteins, which then recruit and activate caspase‐1. Caspase‐1 is toxic to neuronal cells by cleaving pro‐IL‐1β and pro‐IL‐18 into mature proinflammatory cytokines. IL‐1 receptor antagonists are protective in animal models of stroke and in phase II clinical trials.^312^ The NLRP3 inflammasome drives the inflammatory response after the transient intermediate phase of IRI and NLRP3 inflammasome continues to drive neuroinflammation within the subacute stroke phase.^313^ Inhibition of NLRP3 inflammasome can reduces neurological deficits and long‐term cognitive impairment, and reduces infarcts. MCC950 also reduced brain damage in rats.^314^, ^315^ In intracerebral hemorrhage models and cerebral infarction controlled cortical impingement models, inflammasome inhibitors that covered all inflammasomes or specific NLRP3 significantly attenuated inflammatory responses and reduced infarct volume, accompanied by clear evidence of reduced caspase‐1 activation in mice.^312^ The disease‐driven role of NLRP3 suggest that NLRP3 blockers have therapeutic potential for stroke.

### 5.4 Pyroptosis and ALS

ALS is a motor neuron disease characterized by the degeneration of motor neurons in the motor cortex, brain stem and spinal cord. Studies have found that neuroinflammation plays an important role in its course. The expression of NLRP3, GSDMD, and IL‐1β was detected in microglia in the motor cortex and spinal cord of ALS patients, suggesting that NLRP3 inflammasome was activated in ALS patients. In addition, increased microglial pyroptosis was also found in TDP‐43A315T ALS mice and correlated with neuronal death.^294^ Copper/zinc superoxide dismutase (SOD1) gene mutation can lead to ALS animal model. The expression of caspase‐1 is reduced in in transgenic mice expressing mutant human SOD1 with a substitution of glycine to alanine in position 93 (mSOD1(G93A)), and intraventricular administration of caspase‐1 inhibitor zVAD‐fmk can delay the onset and mortality of ALS.^316^ In addition, SOD1 was found to activate caspase‐1 and IL‐1β, and mutant SOD1 can be recognized by ASC to limit caspase‐1‐mediated inflammation.^317^ As the disease progresses, the expression of GSDMD, NLRP3, activated caspase‐1, and IL‐1β in the spinal cord of ALS mice increases compared with CON mice. In the early stage of ALS, NLRP3, caspase‐1, and IL‐1β are mainly located in ventral horn neurons. In the late stage of ALS, GSDMD, NLRP3, activated caspase‐1, and IL‐1β were mainly expressed in reactive astrocytes and microglia. Activation of NLRP3 inflammasome can lead to pyroptosis of ventral horn neurons in ALS patients, which may be involved in motor neuron degeneration and disease progression in ALS.^318^

### 5.5 Treatment of NDs by targeting pyroptosis

#### 5.5.1 NLRP3 inhibitors

Numerous studies have shown that neuroinflammation plays a key role in neurodegenerative diseases. Studies have shown that the expression of proinflammatory factor IL‐1β is upregulated in the brain of AD patients, and NLRP3 knockout improves behavioral tests in AD mice. Inhibition of NLRP3 by OLT1177 can improve the impairment of learning and memory ability and treat neuroinflammation in AD mice.^319^ Recent studies have shown that fenamic nonsteroidal anti‐inflammatory drugs such as flufenamic acid and mefenamic acid can inhibit cyclooxygenase to exert anti‐inflammatory effects and inhibit NLRP3 activation. This dual anti‐inflammatory fenamic acid has been shown to exert neuroprotective effects in animal models of AD. MCC950 is a widely recognized NLRP3 specific inhibitor and has no effect on other inflammasomes such as NLRP1, NLRC4, and AIM2. The specificity of MCC950 makes it unable to completely block the release of proinflammatory factors in vivo and maintain part of the body's immune response. In an experimental autoimmune EAE mice model, MCC950 ameliorated the disease by inhibiting NLRP3 inflammasome activation by blocking ASC oligomerization. Moreover, tivantinib can also effectively alleviate EAE in mice directly blocking ATPase activity of NLRP3.^320^ The development of antagonists of pyroptosis may transform the treatment of neurodegenerative diseases. Studies have found that Prussian blue nanozyme, a pyroptosis inhibitor, inhibits pyroptosis by inhibiting the activation of NLRP3 inflammasome, which can alleviate motor deficits, rescue dopaminergic neurons, and alleviate the severity of PD in MPTP‐induced PD rats.^286^

#### 5.5.2 Caspase‐1 inhibitors

In addition, inflammasome‐induced pyroptosis may be involved in neuronal death after stroke. AIM2 inflammasome‐mediated pyroptosis may aggravate cognitive impairment after stroke. AIM2 knockout and capase‐1 inhibitor Ac‐YVAD‐CMK treatment significantly improved cognitive function in stroke mice.^321^ In LPS and 6‐OHDA‐induced PD models, caspase‐1 inhibitor Ac‐YVAD‐CMK could protect dopaminergic neurons by inhibiting NLRP3/caspase‐1/IL‐1β signaling pathway. VX‐765, a small molecule inhibitor, can inhibit the expression of caspase‐1 in human microglia and oligodendrocytes, reduce axonal injury, and alleviate neurobehavioral manifestations in mice with immune EAE.^322^ In addition, treatment with caspase‐1 inhibitor VX‐765 also alleviated Zika virus‐induced neuroinflammation and pyroptosis and significantly alleviated nerve damage and brain atrophy in vivo.^323^ VX‐765 has been proved to be an antiepileptic drug with less side effects and has passed clinical phase 2 trials, which has great application prospects. In Febrile seizures (FS) mice, the expression level of caspase‐1 was significantly increased before the onset of the disease. CZL80, an inhibitor of caspase‐1, reduced neuronal excitability to inhibit FS in neonatal mice. CZL80 also reduced epileptic susceptibility in adult mice.^324^ Progressive ischemic stroke is characterized by progressive neurological dysfunction after tissue ischemia. High expression of caspase‐1 can aggravate ischemic brain damage. Caspase‐1 inhibitor CZL80 can inhibit the activation of microglia in the peri‐infarct cortex and promote the recovery of neurological function in stroke mice by inhibiting caspase‐1.^325^ VRT‐018858, a selective caspase‐1 inhibitor, has neuroprotective effects at 1 and 3 h after brain injury and significantly attenuates ischemic injury in rats.^326^ Wuyang Huanwu Decoction (BYHWD) is a classical traditional Chinese medicine used for the treatment of cerebral ischemia. Glycosides are the main effective components of BYHWD against nerve injury. The cerebral I/R model was established by occlusion of the middle cerebral artery for 2 h followed by reperfusion for 24 h. Studies have found that glycosides reduce the protein expression levels of NLRP3, ASC, and caspase‐1, inhibit cell pyroptosis, and play a protective role in neurons.^327^

#### 5.5.3 GSDMD inhibitors

Pyroptosis plays an important role in the development of a variety of nervous system diseases. However, the role of GSDMD, the executor of pyroptosis, in NDs has not been elucidated, but a number of studies have found that inhibiting the expression of GSDMD can greatly help to alleviate the progression of NDs. DMF binds to the cysteine residue of GSDMD to succinylation of GSDMD. Succinylation of GSDMD prevents pyroptosis and thus alleviates EAE in mice.^218^ Sevoflurane has been reported to be neurotoxic, which can lead to cognitive defects in learning and memory during development. NSA and DSF were found to inhibit pore formation in GSDMD. In addition, NSA and DSF treatment also attenuated the release of DAMPs and subsequent plasma membrane disruption induced by sevoflurane challenge, thereby attenuating the neurotoxicity of sevoflurane in vitro.^328^

## 6 PYROPTOSIS AND METABOLIC DISEASES

MDs are caused by metabolic problems, including metabolic disorders and metabolic exuberant causes, mainly including the following diseases: diabetes, obesity, and gout (Table 4).

### 6.1 Pyroptosis and diabetes

Diabetes is a complex metabolic syndrome characterized by hyperglycemia. The NLRP3 inflammasome has been associated with the regulation of glucose homeostasis in rats.^314^ High glucose injury leads to impaired adipose function. STZ induced diabetic myopathy in C57 mice, compared with the control group treated with normal saline, the expression of downstream pyroptosis pathway caspase‐1, IL‐1β, and IL‐18 was significantly upregulated.^329^ In addition, pyroptosis further increases the inflammatory response by releasing the proinflammatory cytokine IL‐6. Hyperglycemia can induce macrophage GSDMD activation and pyroptosis, which plays an important role in the pathogenesis of diabetic periodontal disease, and NLRC4 phosphorylation may play a key role.^330^ Hyperglycemia induces pyroptosis in DCM cells by upregulating miR‐30d.^331^ The cause of hyperglycemia associated with diabetic nephropathy (DN) is inadequate insulin secretion or insulin resistance, which leads to hypoxia and excessive production of inflammatory cytokines. High glucose can increase the expression of TLR4, cleaved caspase‐1, GSDMD‐NT, and the secretion of IL‐1β and IL‐18 in mice diabetic kidneys.^332^, ^333^ TLR4 inhibitor and NF‐κB inhibitor partially reversed the pyroptosis induced by high glucose.^334^ In podocytes stimulated by high glucose, the release of proinflammatory cytokines and chemokines, including intracellular ROS, IL‐6 and IL‐1β, is increased by activation of TLR4/NF‐κB signaling pathway.^173^, ^335^ When duodenojejunal bypass (DJB) surgery was performed in HFD/STZ‐induced diabetic rats, the results showed that pyroptosis was reduced and islet β cells were significantly improved. It is speculated that this may be related to the downregulation of NLRP3 inflammasome signaling in macrophages by DJB surgery.^336^ In addition, it has been confirmed that combined use of trehalose and guavas juice can protect β‐cell function by reducing pyroptosis and protect against diabetic pathological damage in rats.^337^ Pyroptosis has become an important research perspective in the pathogenesis of diabetes, but the mechanism of pyroptosis in diabetes is still unclear.

### 6.2 Pyroptosis and obesity

Obesity is strongly associated with low‐grade inflammation throughout in adipose tissue. The level of adipocyte death is increased significantly in both obese mice and human, and this cell death is due in part to macrophage‐induced adipocyte pyroptosis.^338^, ^339^ Obesity often causes enteric neuronal degeneration, which in turn leads to gastrointestinal motility disorders. It was found that the level of activated caspase‐1 was higher in the myenteric ganglia of obese subjects compared with normal subjects. Mice fed a high‐fat Western diet are prone to degeneration of myenteric nitriergic neurons and colonic motility disorders, which are due to caspase‐11‐mediated pyroptosis.^340^ Metabolic inflammation is a key factor in the pathogenesis of obesity. Cordycepin was found to significantly improve systemic inflammation and body weight gain in mice fed a high‐fat western diet. Further studies have shown that cordycepin can inhibit intestinal oxidative stress injury and reduce intestinal epithelial cell apoptosis and pyroptosis.^341^ Obesity also promotes the assembly of the NLRP3 inflammasome in macrophages, which induces macrophage‐mediated T cell activation and IFN‐γ release.^342^ The NLRP3 inflammasome can regulate adiposity and insulin sensitivity, indicating that glucose homeostasis is improved in mice lacking NLRP3.^343^, ^344^ In obese mice and human, hypertrophic adipocytes may induce obese adipocyte death through pyroptosis‐mediated NLRP3‐dependent caspase‐1 activation.^345^ Obesity is often associated with metabolic infiltration in multiple tissues, and monocytes from obese individuals often exhibit elevated inflammatory caspase activity. The saturated fatty acid can activate monocyte pyroptosis through caspase‐4/5, leading to the release of inflammatory mediators and inflammasome activation.^346^

### 6.3 Pyroptosis and gout

Gout, the autoinflammatory diseases, is characterized by episodes of inflammation, causing fever, and severe joint pain and swelling. The development of gout or pseudogout caused by the deposition of monosodium urate (MSU) or crystals in joints.^347^ There is still a lack of effective treatment strategies. Instead, research has focused on identifying risk factors for disease, and the cellular mechanisms by which crystals trigger inflammatory responses. Gout is associated with high levels of NLRP3, caspase‐1, IL‐1β, and IL‐18 detected in serum and synovial fluid of patients.^348^ Bromodomain‐containing protein 4 (BRD4) has been reported to mediate the regulation of NF‐κB signaling through acetylation of RELA13. BDR4 inhibitor JQ‐1 improves rheumatoid arthritis by blocking NF‐κB activation. In addition, the BRD4 inhibitor JQ‐1 was effective in reducing joint swelling and synovial inflammation in MSU‐induced rat model. Exploring the mechanism showed that BRD4 was involved in MSU induced gouty arthritis by regulating the NF‐κB/NLRP3/GSDMD signaling pathway. NLRP3, IL‐1β are risk factors for mice gout.^349^ The expression of NLRP3 and IL‐1β in PBMCs was significantly increased.^350^ GLUT1‐mediated glucose uptake is instrumental during NLRP3 activation induced by MSU and calcium pyrophosphate crystals.^351^ Raf kinase inhibitor protein (RKIP) has been found to inhibit the activation of NLRP1, NLRP3, and NLRC4 inflammasomes. In bone marrow‐derived macrophages, RKIP deficiency activates NLRP1, NLRP3, and NLRC4 inflammasomes. Mechanistically, RKIP directly binds to ASC to interrupt inflammasome activation. Deletion of RKIP can aggravate inflammasome‐related diseases, such as MSU‐induced gouty arthritis and HFD‐induced metabolic disorders.^352^ Signaling resulting from inflammasome activation is a very effective approach to treat gout.

### 6.4 Treatment of metabolic diseases by targeting pyroptosis

#### 6.4.1 NLRP3 inhibitors

The abnormal activation of NLRP3 inflammasome is closely related to the occurrence and development of a variety of abnormalities, such as CAPS, type 2 diabetes, AD, and so on. A specific NLRP3 inhibitor, MCC950, was found to reduce the severity of DN.^329^ In addition, CY‐09 could directly bind to the ATP in the NACHT domain of NLRP3 and inhibit activation of the NLRP3 inflammasome; thus, relieving the symptoms of type 2 diabetic mice.^353^ Oridonin is a Chinese herbal medicine component that has been reported to have anti‐inflammatory and antitumor effects. Recent studies have shown that oridonin directly binds to the NACHT domain of NLRP3 and specifically inhibits the activation of NLRP3 inflammasome, thereby alleviating gouty arthritis and type 2 diabetes in mice.^202^ Several NLRP3 inhibitors have been shown to alleviate gout by directly inhibiting NLRP3 inflammasome activation. For example, metformin and resveratrol can reverse the damage process and protect mitochondrial integrity by limiting ER stress to prevent NLRP3 inflammasome activation in high‐fat mice.^354^, ^355^ Erianin alleviates gout by directly interacting with NLRP3 to inhibit NLRP3 assembly. At the cellular level, artemisinin inhibited MSU‐induced NEK7 and NLRP3 expression, thereby reducing symptoms such as ankle swelling in arthritic mice. In addition, sulforaphane alleviated MSU‐induced arthritis symptoms and inflammatory cell infiltration in gout mice by directly inhibiting NLRP3 inflammasome activation. Eucalyptol inhibits gout and joint inflammation mainly by inhibiting NLRP3 inflammasome activation and proinflammatory cytokine production through antioxidant mechanism. DSF inhibits NLRP3 activation by inhibiting mitochondrial ROS production and has a significant effect on MSU‐induced gout inflammation. Gallic acid is an active phenolic acid that has been shown to have anti‐inflammatory effects. Gallic acid was found to inhibit MSU‐induced recruitment of neutrophils and macrophages to the synovial region of the joint. Gallic acid also inhibits the production of ROS, thereby limiting the activation of NLRP3 inflammasome and pyroptosis, thereby playing a protective role in the mouse gouty arthritis model.

#### 6.4.2 Caspase‐1 inhibitors

It is now generally accepted that chronic tissue inflammation is the main cause of insulin resistance and metabolic dysfunction in obese patients. Caspase‐1 plays an important role in pyroptosis after inflammasome activation and the release of inflammatory factors. Therefore, inhibiting caspase‐1 to reduce inflammation and pyroptosis may be an important method to alleviate metabolism‐related diseases. Leiden mice obesity was induced by HFD. Treatment with Ac‐YVAD‐cmk inhibited weight gain and dyslipidemia in mice. Treatment with caspase‐1 inhibitor Ac‐YVAD‐cmk reduced the development of liver fibrosis and insulin resistance in obesity complications.^356^ VX‐765 is a broad‐spectrum caspase‐1 inhibitor, which can inhibit pyroptosis and the release of inflammatory factors by inhibiting caspase‐1, thereby alleviating a variety of inflammatory diseases, such as multiple sclerosis,^322^ silicosis,^357^ and atherosclerosis,^208^ and is expected to relieve inflammation related to MDs.

#### 6.4.3 GSDMD inhibitors

Pyroptosis plays a crucial role in the disease progression of gouty arthritis and has become a target because of its clinical therapeutic potential. Obesity can also lead to changes in the structure and function of the heart, which seriously threatens human life. Wogonin treatment can alleviate obesity‐induced lipid metabolism disorders and cardiac injury by inhibiting pyroptosis and IL‐17 signaling pathway. It alleviates myocardial pyroptosis, myocardial injury, and lipid metabolism disorder in mice induced by HFD. Diabetes can cause a variety of complications, among which diabetic foot is the most common. Inhibition of GSDMD by persulfiram can inhibit the formation of NETs and accelerate the wound healing of diabetic foot.^358^ Studies have found that when obesity occurs, GSDMD interacts with interferon regulatory factor 7 and forms a complex to promote adipocyte pyroptosis. By targeting GSDMD, MT inhibits the activation of NLRP3 inflammasome in adipose tissue of mice and inhibits pyroptosis to alleviate obesity.^359^ NSA, as a GSDMD‐targeted inhibitor, can inhibit pyroptosis by inhibiting GSDMD, thereby alleviating a variety of inflammatory diseases, such as inflammatory bowel disease,^360^ acute liver failure,^361^ and pulmonary fibrosis^362^ and is expected to alleviate inflammation related to MDs.

## 7 CONCLUSIONS AND PERSPECTIVE

Pyroptosis is a new type of inflammatory PCD that is triggered by inflammatory caspase and gasdermins proteins.^363^ To date, pyroptosis is mainly mediated by four signaling pathways, namely canonical pyroptosis pathway, noncanonical pyroptosis pathway, other caspases‐mediated pyroptosis pathway, and granzymes and other proteases‐mediated pyroptosis pathway. Increasing studies on pyroptosis show a great progress in extensively various diseases; therefore, we write the review to summarize recent developments in understanding of the complex mechanism of pyroptosis and application in different diseases (CVDs, cancer, NDs, and MDs). As described above, the evidence has verified that pyroptosis plays an important role and is closely related to the occurrence and development of diseases (CVDs, cancer, NDs, and MDs). There are many factors causing various diseases, among which cell death is one of the most important causes. However, the mechanisms that trigger cell death remains unclear.

In the past decade, emerging evidence has demonstrated that pyroptosis was found in CVDs, cancer, NDs, and MDs, which are involved in the pathogenesis processes of many diseases. In review, we summarize current insights into the complicated relationship between pyroptosis and CVDs, cancer, NDs, and MDs, and also discusses a promising new strategy for treating these diseases by targeting pyroptosis. Since pyroptosis contributes to the development of CVDs, cancer, NDs, and MDs, inhibition of pyroptosis is a promising and effective strategy for the treatment of these diseases. We also summarize current insights into the complicated relationship between pyroptosis and CVDs, cancer, NDs, and MDs, discuss the promising new strategy for treating these diseases by targeting pyroptosis and its upstream inflammasome.

In conclusion, the pathogenic mechanism, and functions of pyroptosis underlying the occurrence, development, and outcome of related diseases are still to be investigated. Such a deep investigation of the relationship between related diseases and pyroptosis is the focus of relevant research fields, providing new clinical approaches for the treatment of various diseases.

## AUTHOR CONTRIBUTION

Xiangyu Jin, Yinchu Ma, and Didi Liu wrote and drafted the manuscript and figures. Yi Huang designed and revised the manuscript. All authors contributed to the article and approved the submitted version.

## ETHICS STATEMENT

Not applicable.

## CONFLICT OF INTEREST STATEMENT

The authors declare no competing interests.

## Data Availability

The primary data for this study are available from the authors upon request.
